# Supplementary material for: Early Pandemic Evaluation and Enhanced Surveillance of COVID-19 (EAVE II): protocol for an observational study using linked Scottish national data
Source: BMJ Open. 2020 Jun 21;10(6):e039097. doi: 10.1136/bmjopen-2020-039097 (PMC7311023; doi:10.1136/bmjopen-2020-039097)
Supplement: Author's manuscript [file bmjopen-2020-039097.draft_revisions.pdf]

BMJ Open

BMJ Open is committed to open peer review. As part of this commitment we make the peer review history of every article we publish publicly available.

When an article is published we post the peer reviewers' comments and the authors' responses online. We also post the versions of the paper that were used during peer review. These are the versions that the peer review comments apply to.

The versions of the paper that follow are the versions that were submitted during the peer review process. They are not the versions of record or the final published versions. They should not be cited or distributed as the published version of this manuscript.

BMJ Open is an open access journal and the full, final, typeset and author-corrected version of record of the manuscript is available on our site with no access controls, subscription charges or pay-per-view fees (http://bmjopen.bmj.com).

If you have any questions on BMJ Open's open peer review process please email info.bmjopen@bmj.com

BMJ Open

Early pandemic evaluation and enhanced surveillance of COVID-19 (EAVE II): protocol for an observational study using linked Scottish national data

Journal:	*BMJ Open*
Manuscript ID	bmjopen-2020-039097
Article Type:	Protocol
Date Submitted by the Author:	06-Apr-2020
Complete List of Authors:	Simpson, Colin; Victoria University of Wellington, Wellington School of Health, Faculty of Health; The University of Edinburgh, Usher Institute<br>Robertson, Chris; University of Strathclyde, Department of Mathematics and Statistics; Public Health Scotland<br>Vasileiou, Eleftheria; University of Edinburgh, Usher Institute ;<br>McMenamin, Jim; Public Health Scotland<br>Gunson, Rory; West Of Scotland Specialist Virology Centre<br>Ritchie, Lewis; University of Aberdeen, Centre of Academic Primary Care<br>Woolhouse, Mark; The University of Edinburgh, Usher Institute<br>Morrice, Lynn; The University of Edinburgh, Usher Institute<br>Kelly , Dave; Albasoft Ltd, The Centre for Health Science<br>Stagg, Helen R.; The University of Edinburgh, Usher Institute<br>Marques, Diogo; Public Health Scotland<br>Murray, Josie; Public Health Scotland<br>Sheikh, Aziz; The University of Edinburgh, Usher Institute
Keywords:	Public health < INFECTIOUS DISEASES, Epidemiology < INFECTIOUS DISEASES, PUBLIC HEALTH, RESPIRATORY MEDICINE (see Thoracic Medicine)

SCHOLARONE™
Manuscripts

I, the Submitting Author has the right to grant and does grant on behalf of all authors of the Work (as defined in the below author licence), an exclusive licence and/or a non-exclusive licence for contributions from authors who are: i) UK Crown employees; ii) where BMJ has agreed a CC-BY licence shall apply, and/or iii) in accordance with the terms applicable for US Federal Government officers or employees acting as part of their official duties; on a worldwide, perpetual, irrevocable, royalty-free basis to BMJ Publishing Group Ltd ("BMJ") its licensees and where the relevant Journal is co-owned by BMJ to the co-owners of the Journal, to publish the Work in this journal and any other BMJ products and to exploit all rights, as set out in our licence.

The Submitting Author accepts and understands that any supply made under these terms is made by BMJ to the Submitting Author unless you are acting as an employee on behalf of your employer or a postgraduate student of an affiliated institution which is paying any applicable article publishing charge ("APC") for Open Access articles. Where the Submitting Author wishes to make the Work available on an Open Access basis (and intends to pay the relevant APC), the terms of reuse of such Open Access shall be governed by a Creative Commons licence – details of these licences and which Creative Commons licence will apply to this Work are set out in our licence referred to above.

Other than as permitted in any relevant BMJ Author's Self Archiving Policies, I confirm this Work has not been accepted for publication elsewhere, is not being considered for publication elsewhere and does not duplicate material already published. I confirm all authors consent to publication of this Work and authorise the granting of this licence.

For peer review only

Early pandemic evaluation and enhanced surveillance of COVID-19 (EAVE II): protocol for an observational study using linked Scottish national data

Prof. Colin Simpson, Wellington School of Health, Faculty of Health, Victoria University of Wellington, Wellington, New Zealand and Usher Institute, The University of Edinburgh, Edinburgh, UK

Prof. Chris Robertson, Department of Mathematics and Statistics, University of Strathclyde, Glasgow, UK and Public Health Scotland, Glasgow, UK

Dr. Eleftheria Vasileiou, Usher Institute, The University of Edinburgh, Teviot, Medical School, Edinburgh, UK, EH8 9AG, eleftheria.vasileiou@ed.ac.uk, +44 07732961139

Dr. Jim McMenamin, Public Health Scotland, Meridian Court, 5 Cadogan Street, Glasgow, UK

Prof. Rory Gunson, West of Scotland Specialist Virology Centre, Glasgow, UK

Sir Lewis D. Ritchie, Centre of Academic Primary Care, University of Aberdeen, Aberdeen, UK

Prof. Mark Woolhouse, Usher Institute, The University of Edinburgh, Edinburgh, UK

Dr. Lynn Morrice, Usher Institute, The University of Edinburgh, Edinburgh, UK

Dave Kelly, Albasoft Ltd, The Centre for Health Science, Raigmore, Inverness, UK

Dr. Helen R. Stagg, Usher Institute, The University of Edinburgh, Edinburgh, UK

Diogo Marques, Public Health Scotland, Meridian Court, 5 Cadogan Street, Glasgow, UK

Dr. Josie Murray, Public Health Scotland, Meridian Court, 5 Cadogan Street, Glasgow, UK

Prof. Aziz Sheikh, Usher Institute, The University of Edinburgh, Edinburgh, UK

Keywords: COVID-19, coronavirus, pandemic, surveillance, serology

Word count: 3,082

For peer review only

For peer review only - http://bmjopen.bmj.com/site/about/guidelines.xhtml

ABSTRACT

Introduction

Following the emergence of the novel coronavirus SARS-CoV-2 in December 2019 and the ensuing COVID-19 pandemic, population-level surveillance and rapid assessment of the effectiveness of existing or new therapeutic or preventive interventions are required to ensure that interventions are targeted to those at highest risk of serious illness or death from COVID-19. We aim to re-purpose and expand an existing pandemic reporting platform to determine the attack rate of SARS-CoV-2, the uptake and effectiveness of any new pandemic vaccine (once available), and any protective effect conferred by existing or new antimicrobial drugs and other therapies.

Methods and analysis

A prospective observational cohort will be used to monitor daily/weekly the progress of the COVID-19 epidemic and to evaluate the effectiveness of therapeutic interventions in approximately 5.4 million individuals registered in general practices across Scotland. A national linked dataset of patient-level primary care data, out-of-hours, hospitalisation, mortality and laboratory data will be assembled. The primary outcomes will measure association between: a) laboratory confirmed SARS-CoV-2 infection, morbidity and mortality and demographic, socioeconomic and clinical population characteristics; and b) healthcare burden of COVID-19 and demographic, socioeconomic and clinical population characteristics. The secondary outcomes will estimate: a) the uptake (for vaccines only); b) effectiveness; and c) safety of new or existing therapies, vaccines and antimicrobials against SARS-CoV-2 infection. The association between population characteristics and primary outcomes will be assessed via multivariate logistic regression models. The effectiveness of therapies, vaccines and antimicrobials will be assessed from time-dependent Cox models or Poisson regression models. Self-controlled study designs will be explored to estimate the risk of therapeutic and prophylactic-related adverse events.

Ethics and dissemination

We obtained approval from the National Research Ethics Service Committee, Southeast Scotland 02. The study findings will be presented at international conferences and published in peer-reviewed journals.

For peer review only

For peer review only - http://bmjopen.bmj.com/site/about/guidelines.xhtml

Strengths and limitations of this study

- We plan to interrogate national data on the Scottish general population.
- We are expanding an existing national pandemic reporting platform, which uses anonymised individual patient-level data from general practices, hospitals, death registry, virology (reverse transcriptase polymerase chain reaction RT-PCR) and serology tests to investigate the epidemiology of COVID-19 and assess the effectiveness of existing or future preventive and treatment measures.
- This is an observational study using routinely collected data. Insufficient adjustment for confounding, either due to insufficiently granular variable measurement or a lack of variable measurement is a potential concern. The direction of likely bias will be described in all cases.

For peer review only

For peer review only - http://bmjopen.bmj.com/site/about/guidelines.xhtml

INTRODUCTION

In the last two centuries, six pandemics (global epidemics) have emerged due to novel influenza and coronavirus strains. During the 20th century, influenza caused three pandemics (1918-19, 1957-58, 1968-69), resulting in millions of clinical cases and deaths.[1-4] An estimated 20-50 million deaths were reported during the 1918-19 influenza pandemic. Fewer (between 1-4 million deaths) were estimated for the 1957-58 and 1968-69 influenza pandemics, respectively.[1-4] The high mortality rates observed in the 20th century against the H1N1, H2N2 and H3N2 influenza viruses were mainly due to lack of prophylactic and therapeutic interventions, such as influenza vaccines and anti-viral medications.[1-4] By comparison, the first pandemic of the 21st century arose from a novel coronavirus, severe acute respiratory syndrome (SARS-CoV), which emerged in 2002-03.[5] SARS caused more than 8,000 infections and 700 deaths globally.[2, 5] In 2009-10, the fourth recorded influenza pandemic due the influenza A (H1N1) subtype emerged in Mexico, resulting in more than 200,000 deaths globally and more than a third of the gloabal population infected.[2] Previous exposure to seasonal influenza vaccination induced little or no cross-reactive antibody responses.[6] Particularly low immunological protection against the virus was observed in the younger population (<30 years old) compared to older adults.[6]

In December 2019, a novel coronavirus-SARS coronavirus 2 (SARS-CoV-2)- emerged in Wuhan, China.[7-8] In the space of four months, this virus has now spread globally. The World Health Organization (WHO) declared the coronavirus outbreak a Public Health Emergency of International Concern on 30 January 2020 and then a pandemic on 11 March 2020, as a result of the worldwide spread of the COVID-19 disease.[8] As of 3 April 2020, the WHO has reported more than 970,000 confirmed infections globally and over 50,000 deaths.[8] The elderly, people with underlying medical conditions and people with poor immune function and long-term users of immunosuppressive agents are particularly vulnerable to SARS-CoV-2 and at risk of severe coronavirus-related illness.[7-10] Current data indicate that SARS-CoV-2 has a lower mortality rate, ranged between 0.25% to 3% (despite the high number of deaths), than for SARS-CoV (10%) and Middle East Respiratory Syndrome-related coronavirus (MERS-CoV) (37%), respectively.[11-12] It has been postulated (using data from case-studies) that the main driver of disease severity amongst younger patients for COVID-19 are immunopathological lesions, resulting from an excessive pro-inflammatory host response or cytokine storm.[13-14] Amongst older people, an impaired interferon pathway and systemic virus dissemination beyond the respiratory tract may lead to severe disease.[13-14] The absence of immunity from historic exposure to existing seasonal vaccination or anti-viral therapy also (in comparison to influenza) renders COVID-19 a significant global health threat, which demands an urgent response from national and international agencies.

Rapid large observational epidemiological studies are now required to identify the epidemiological and clinical profile of the COVID-19 pandemic. These studies can also be used to estimate the effectiveness of any existing or new healthcare interventions, such as vaccines and anti-viral therapies (e.g. the introduction of any new pandemic vaccine), where it is unethical and/or not feasible to mount more rigorous experimental studies.

For peer review only - http://bmjopen.bmj.com/site/about/guidelines.xhtml

Using linked routine sources of primary, secondary, mortality and virological/serological testing data, this study aims to describe the epidemiology of COVID-19 in Scotland and in due course help establish the effectiveness of existing or new therapeutic interventions against the coronavirus that are not subjected to formal clinical trials. Specifically, our objectives are to:

Primary objectives
a) Determine the epidemiological risk factors for infection, morbidity, mortality of COVID-19 (e.g. laboratory and serology confirmed SARS_CoV-2 infection in relation to demographic, socioeconomic and clinical population characteristics);

b) Determine the healthcare burden of COVID-19 (e.g. COVID-19-related morbidity and mortality in relation to demographic, socioeconomic and clinical population characteristics);

Secondary objectives
a) Measure the uptake of prophylactic interventions (e.g. vaccines);

b) Estimate the effectiveness of any new or existing of prophylactic and therapeutic interventions (e.g. new or repurposed therapies, vaccines, antimicrobials);

c) Assess the safety of any new or existing of prophylactic and therapeutic interventions (e.g. new or repurposed therapies, vaccines, antimicrobials).

This work will re-purpose and expand the hibernated Early Estimation of Vaccine and Anti-Viral Effectiveness (EAVE) project part of the NIHR Pandemic Preparedness Research Portfolio [15][16], and a proven platform for studies on seasonal and pandemic influenza vaccine and anti-viral assessment.[16-20]

METHODS

Study design and population

We will undertake a timely analysis of a large national open prospective observational cohort of patients using a unique community, hospital and laboratory linked dataset. We will seek to extract data on 5.4 million people from across Scotland (Figure 1).

Databases

Individual-level data from general practices will be extracted and linked deterministically to secondary and laboratory healthcare datasets using the Community Health Index (CHI).[16] The CHI number is a unique identifier provided by the National Health Service (NHS) for each resident in Scotland registered with a general practice. The linkage of the datasets and analysis will take place within a secure Trusted Research Environment (TRE).[16]

Primary care
Almost all individuals in Scotland are registered with a general practice, which provide free of charge healthcare services. Data from all patients registered in general practices will be extracted and studied. The University of Edinburgh and Public Health Scotland (PHS) will recruit the additional general practices through Albasoft Ltd.[16-20] Albasoft Ltd is the trusted

For peer review only - http://bmjopen.bmj.com/site/about/guidelines.xhtml

third party that will carry out the data extraction from all general practices using the Enhanced Services Contract Reporting Options (ESCRO) system.[16-20] We will also extract data from a network of COVID-19 Community Hubs and Assessment Centres established by NHS Health Boards across Scotland.[21] The aim of this network is to provide a direct and rapid route of people with COVID-19 symptoms that have worsened or not improved after a week. Patients can call NHS 24 for an initial assessment and then if needed the call will be passed to a telephone Community Hub, staffed by clinical decision makers.[21] The clinical decision maker will then decide if an appointment for a face to face consultation at an Assessment Centre is necessary.[21]

Secondary care

The Scottish Morbidity Record (SMR) database will be used to derive information for all in-patient hospitalisations and emergency admissions in Scotland, which is maintained by the Information Services Division (ISD).[22] Specifically, we will use data from the SMR01 record which is an episode-based patient record for all inpatients and day cases discharged from non-obstetric and non-psychiatric specialties in Scotland.[23] Data from the SMR02 record will also be used, which is an episode based patient record for all inpatients and day cases from Obstetric specialties in the NHS Scotland.[24] The SMR dataset also contains mortality data which derive from the National Records of Scotland (NRS).[25] Regular validation checks are applied to the SMR database. The latest data quality assessment of these SMR datasets have shown over 90% completeness and accuracy in consistency with previous years.[26] We will also extract and link data on prescribing and administration of medicines for inpatients which are available from Scottish Hospital Electronic Prescribing and Medicines Administration (HEPMA) systems.[27] The study data will also be linked with data from patients admitted to adult general Intensive Care Units (ICU) which derive from the Scottish Intensive Care Society Audit Group (SICSAG) national database.[28] The database contains detailed information on the management of critically ill or injured patients. Data are collected from all general ICU and combined ICU//High Dependency Units (HDU). Data from more than 90% general HDUs and a number of specialist ICUs and HDUs are collected by the database.[28]

Laboratory and serology data

The Electronic Communication of Surveillance in Scotland (ECOSS) system of PHS is a database that holds surveillance data on various microorganisms (e.g. influenza virus, coronavirus) and infections reported from diagnostics and reference laboratories.[29] Data on laboratory results for all reverse transcriptase polymerase chain reaction (RT-PCR) tests carried out in Scotland are being collated by ECOSS and can be linked to other data sources).[29] Positive laboratory swab samples for SARS-CoV-2 will also be sent to national sequencing centres where 500 SARS-CoV-2 genome sequences will be performed.

In a sub-study, the West of Scotland Specialist Virology Laboratory will collect and store residual sera from routine blood tests from patients until the serology test becomes available.[30] The EAVE study has already stored 1,000 biochemistry samples from a subset of participating practices from 2014, demonstrating that a potential mechanism for the collection and storage of the residual sera work.[16] We aim to collect and store serially

For peer review only - http://bmjopen.bmj.com/site/about/guidelines.xhtml

throughout the duration of the COVID-19 pandemic. This will be used to determine exposure to SARS-CoV-2 and other viruses by the presence of antibodies).[16]

Exposure definitions and potential confounding factors

The following exposure variables will be used in relation to study's primary outcomes: sex, age, socioeconomic status (SES) and clinical at-risk group. SES will be determined based on the Scottish Multiple Deprivation Index (SIMD). The SIMD classification is based on deprivation quintiles. Quintile 1 refers to the most deprived and quintile 5 refers to the least deprived. The SIMD is a combination of 38 indicators of the following seven domains: income, employment, health, education, housing, geographical access to services and crime.[20] Clinical at-risk groups refer to individuals with certain underlying medical conditions where are at-risk of COVID-related complications and for whom seasonal influenza vaccination is recommended. The following clinical at-risk conditions will be considered: a) chronic respiratory disease (with chronic obstructive pulmonary disease and asthma as subsets); b) chronic heart disease; c) chronic liver disease; d) chronic kidney disease; e) chronic liver disease; f) chronic neurological disease; g) diabetes; h) conditions or medications causing impaired immune function; i) pregnancy; j) asplenia or dysfunction of spleen; k) obesity (body mass index (BMI) < 20, 20-25, 25-30, 30-39, ≥ 40 kg/m^2) l) hypertension (subsets controlled/uncontrolled hypertension), and m) multimorbidity .[20] This list will be updated as more evidence arises within the medical literature. The following exposure variables will be used in relation to study's secondary outcomes: any new vaccines against SARS-CoV-2 and existing or new therapies and antimicrobial medication against COVID-19. These will be determined once our study data are available and any new therapies, vaccines and antimicrobials specifically against the SARS-CoV-2 virus have been produced.

A number of aforementioned and additional population characteristics below will also be used as potential confounding factors in relation to the study's primary and secondary outcomes. Charlson Comorbidity Index will represent the weighted comorbidity score based on secondary care data.[16-20] The urban/rural location will be determined based on the urban/rural 8 fold classification (UR8). The UR8 is the definition of rural areas in Scotland; 1 is assigned to large urban areas and 8 is assigned to remote rural areas.[20] Smoking status will be determined and presented into the following four categories: Current smoker, non-smoker, ex-smoker and not recorded for patients with no data on smoking.[16-20] Previous healthcare usage will be used to measure number of primary care consultations and secondary care admissions in previous years. The number of prescriptions will also be determined for previous years.[16-20] General practice will also be used to account the effect of clustering within practices.

Outcome definitions

The primary outcomes of this study will include: a) laboratory confirmed SARS-CoV-2; b) serum from blood samples taken from biochemistry tests (or rapid antibody tests if available) will be used to determine exposure to SARS-CoV-2 infection by the presence of antibodies; and c) SARS-CoV-2 infection related clinical outcomes including general practice, COVID centres and out-of-hours consultations, hospital admissions, emergency admissions, out of hours consultations and deaths. Secondary outcomes include: a) vaccine uptake proportions; b)

For peer review only - http://bmjopen.bmj.com/site/about/guidelines.xhtml

prevention and reduction of SARS-CoV-2 infection-related general practice consultations, hospital admissions, emergency admissions, out of hours consultations and deaths due to therapies, vaccines and antimicrobials; and c) adverse events related to therapies – e.g. vaccine, antimicrobial administration or other therapies.

Statistical analysis

Baseline characteristics of all study participants will be described in relation to the study's exposures and outcomes of interest. Mean, median, proportions, odds ratios (ORs) and rate ratios (RRs), together with a measure of dispersion will be provided where appropriate to describe differences between the various study groups based on the nature of each variable. The amount of missing data will be described for each variable. Two-tailed hypotheses tests with a 5% significance level will be used for all study's outcomes. All analyses will be carried out using the R statistical programming language.[16-20]

Primary analyses

Epidemiology and healthcare burden of COVID-19

We will determine the epidemiological risk factors such as demographic, socioeconomic and clinical population characteristics in relation to laboratory and serology confirmed SARS-CoV-2 infection. The healthcare burden of COVID-19 in terms of morbidity and mortality in relation to to demographic, socioeconomic and clinical population characteristics will also be determined. SARS-CoV-2 infection will be confirmed via laboratory (RT-PCR) and serology testing. Healthcare burden will be measured via general practice consultations, out-of-hours consultations, A&E attendances and hospital admissions. Exposure of interest as per our objectives a and b will change over time as the medical literature and surveillance reporting is continuously updated. Currently, particularly factors of interest for Scotland include: age; sex, geographical location, socioeconomic status, underlying condition or medication and BMI. Analytical techniques including descriptive analysis, univariable and multivariable logistic regression will be used to determine the association between different exposure variables and the likelihood (odds) of the study's primary outcomes (SARS-CoV-2 infection, morbidity, mortality and healthcare burden). The effect of confounders and effect modifiers will be explored through causal frameworks generated for each hypothesis,[31] with clinical input.

Secondary analyses

Vaccine uptake

Differences in vaccine uptake will be measured in relation to demographic, socioeconomic and clinical population characteristics. As per primary analyses, exposure of interest will change over time as the medical literature and surveillance reporting is continuously updated. Key sociodemographic and clinical factors will be analysed including age, sex, socioeconomic status and underlying condition. Analytical techniques including univariable and multivariable logistic regression will be used to determine the association between different exposure variables and vaccine uptake. The effect of confounders and effect modifiers will be explored through causal frameworks generated for each hypothesis,[31] with clinical input. Key confounding factors will include age, sex, socioeconomic status and underlying condition.

For peer review only - http://bmjopen.bmj.com/site/about/guidelines.xhtml

Effectiveness of new or existing prophylactic and therapeutic interventions

We will assess the effectiveness of any new or repurposed therapies, vaccines and antimicrobials against SARS-CoV-2-related morbidity and mortality such as general practice and out of hours consultations, hospitalisations, emergency admissions and deaths. Exposure of interest (therapies, vaccines and antimicrobials) will change over time as the medical literature and surveillance reporting is continuously updated. The proportion of SARS-CoV-2-related clinical outcomes and deaths will be estimated between vaccinated and unvaccinated cases. Vaccine effectiveness (VE) and 95% CIs will be calculated using the formula, VE = (1-Risk Ratio)*100 for unadjusted and adjusted VE estimates. A time-dependent Cox model or the equivalent Poison regression models (taking into account the time at risk and the possibility of multiple events (not for death)) will provide the RRs and 95% CIs of VE for prevention of SARS-CoV-2 related clinical outcomes and deaths. Causal frameworks will be generated for each hypothesis,[31] with clinical input. Key confounders for the VE models will include age, sex, socioeconomic status and underlying condition, with vaccination group representing a time-dependent covariate. In these VE models, propensity variables related to vaccine receipt and effect modifiers (e.g. vaccinations, consultations and hospitalisation in the previous season, urban/rural status, smoking status, Charlson Score and pregnancy) will be used to control for the healthy vaccine effect.[16] This is in addition to the demographic variables, which will always be used.

Similar statistical methods will be used to assess the protective effects of therapies and antimicrobials. A binary variable of ever/never exposure to therapies/antimicrobials as an explanatory variable will be included in the VE analyses. The therapy/antimicrobial exposure will be a second time-dependent exposure for consultation, hospitalisation and death rates analysis. We will also consider using a measure of the volume of therapy/antimicrobial exposure (e.g. length or dose of prescription) if the data are adequate. Use of therapies/antimicrobials will be included as a covariate in any of our models where primarily assess VE. Alternatively, exposure to the vaccine will be included in any of our models where primarily assess the effect of therapies/antimicrobials, if appropriate. For example, the effect of therapies/antimicrobials may be assessed from a period before the vaccine becomes available and, in such instances, no adjustment needs to be made.

Safety of new or existing prophylactic and therapeutic interventions

We will determine any adverse events following the administration of new or repurposed therapies, vaccines and antimicrobials. Specific therapies, vaccines and antimicrobials against SARS-CoV-2 will be determined as the outbreak unfolds and depending on existing medical literature. The risk of adverse events will be estimated using self-controlled study designs. The main assumption in these study designs is that in case of an adverse event related to prophylactic and therapeutic agent exposure then the occurrence of on adverse event in the period after administration is greater than in periods in the same patients that are temporally not related to prophylactic and therapeutic agent administration.[20] The risk interval (the period at risk for an adverse outcome) and the control interval (the period not at risk for an adverse outcome) will be determined separately for each outcome.[20] Causal frameworks will be generated for each hypothesis,[31] with clinical input. The main advantage of the self-

For peer review only - http://bmjopen.bmj.com/site/about/guidelines.xhtml

controlled designs is the control for all fixed individual-level confounding since any comparisons are carried out for the same individual rather than between exposed and unexposed populations to therapies, vaccines or antimicrobials.[20] Key confounding and effect modifiers will be determined as the outbreak unfolds and depending on existing medical literature.

Sample size

Our prospective cohort will be constructed from patients registered in all general practices across Scotland with a combined list size of 5.4 million people of all ages. Sample size calculations to assess vaccine and antiviral effectiveness against pandemic influenza have been provided in previous work [16]. Similar sample size calculations are likely to be applicable to the current COVID-19 pandemic, however, sample size calculations (one per key analysis) are dependent on how the COVID-19 outbreak unfolds in Scotland. Thus, our power to answer each objective will be dependent on the frequency of the relevant outcome. Power calculations will be carried out subsequent to the first wave of the pandemic.

Ethics and dissemination

National Research Ethics Service Committee, South East Scotland 02. Findings from this study will be presented at international conferences and published in peer-reviewed journals. Meta-data produced in this study will also become available to Health Data Research UK (HDRUK) Gateway through BREATHE – The Health Data Research Hub for Respiratory Health. STROBE and RECORD (via the COVID-19 extension) will be used to guide transparent reporting.

Contributors

CRS, CR, JM, LDR, RG and AS contributed to the conception of the study. All authors contributed to the study design. All authors contributed to drafting the protocol. All authors revised the manuscript for important intellectual content. All authors gave final approval of the version to be published.

Acknowledgments

The authors thank and acknowledge Kenny Fraser at Triscribe Ltd for his support in this study.

Funding

The EAVE project was funded by the National Institute for Health Research Health Technology Assessment Programme (project number 13/34/14). EAVE II is funded by the Medical Research Council [MR/R008345/1] and supported by the Scottish Government. We also acknowledge the support of HDR UK. HRS is supported by the Medical Research Council [MR/R008345/1].

Disclaimer

The views and opinions expressed therein are those of the authors and do not necessarily reflect those of the Health Technology Assessment programme, NIHR, NHS or the Department of Health.

Competing interests

None declared.

Provenance and peer review

Not commissioned; externally peer reviewed.

For peer review only - http://bmjopen.bmj.com/site/about/guidelines.xhtml

Patient and Public Involvement

We will pursue the involvement of patients or the public in our research study.

REFERENCES

1. Kilbourne ED. Influenza pandemics of the 20th century. *Emerg Infect Dis* 2006;12(1):9–14.
2. Bloom DE, Cadarette D. Infectious Disease Threats in the Twenty-First Century: Strengthening the Global Response. *Front Immunol* 2019;10:549.
3. Centers for Disease Control and Prevention. Inlfuenza (Flu). Pandemic Influenza. Past Pandemics. Available from: https://www.cdc.gov/flu/pandemic-resources/basics/past-pandemics.html (accessed March 23, 2020).
4. World Health Organization. Influenza. Past Pandemics. Available from: http://www.euro.who.int/en/health-topics/communicable-diseases/influenza/pandemic-influenza/past-pandemics (accessed March 23, 2020).
5. LeDuc JW, Barry MA. SARS, the First Pandemic of the 21st Century. *Emerg Infect Dis* 2004;10(11):e26.
6. Hancock K, Veguilla V, Lu X et al. Cross-reactive antibody responses to the 2009 pandemic H1N1 influenza virus. *N Engl J Med* 2009;361(20):1945-52.
7. Adhikari SP, Meng S, Wu YJ, et al. Epidemiology, causes, clinical manifestation and diagnosis, prevention and control of coronavirus disease (COVID-19) during the early outbreak period: a scoping review. *Infect Dis Poverty* 2020;9(1):29.
8. World Health Organization. Coronavirus disease (COVID-19) outbreak. Available from: https://www.who.int/emergencies/diseases/novel-coronavirus-2019 (accessed March 26, 2020).
9. Guo YR, Cao QD, Hong ZS et al. The origin, transmission and clinical therapies on coronavirus disease 2019 (COVID-19) outbreak - an update on the status. *Mil Med Res* 2020;7(1):11.
10. Yang X, Yu Y, Xu J et al. Clinical course and outcomes of critically ill patients with SARS-CoV-2 pneumonia in Wuhan, China: a single-centered, retrospective, observational study. *Lancet Respir Med* 2020;pii:S2213-2600(20)30079-5. [Epub ahead of print]
11. Wilson N, Kvalsvig A, Barnard LT, et al. Case-fatality estimates for COVID-19 calculated by using a lag time for fatality. *Emerg Infect Dis* 2020;26(6).
12. Wang C, Horby PW, Hayden FG et al. A novel coronavirus outbreak of global health concern. *Lancet* 2020;395(10223):470-473.
13. Guan WJ, Ni ZY, Hu Y et al. Clinical Characteristics of Coronavirus Disease 2019 in China. *N Engl J Med* 2020; [Epub ahead of print]
14. Lescure FX, Bouadma L, Nguyen D et al. Clinical and virological data of the first cases of COVID-19 in Europe: a case series. Lancet Infect Dis 2020;
15. Simpson CR, Beever D, Challen K et al. The UK's pandemic influenza research portfolio: a model for future research on emerging infections. Lancet Infect Dis 2019;19(8):e295-e300.

For peer review only - http://bmjopen.bmj.com/site/about/guidelines.xhtml

16. Simpson CR, Lone N, McMenamin J, et al. Early estimation of pandemic influenza Antiviral and Vaccine Effectiveness (EAVE): use of a unique community and laboratory national data-linked cohort study. *Health Technol Assess* 2015;19(79).
17. Simpson CR, Ritchie LD, Robertson C et al. Vaccine effectiveness in pandemic influenza - primary care reporting (VIPER): an observational study to assess the effectiveness of the pandemic influenza A (H1N1)v vaccine. *Health Technol Asses* 2010;14:313-46.
18. Simpson CR, Ritchie LD, Robertson C et al. Effectiveness of H1N1 vaccine for the prevention of pandemic influenza in Scotland, UK: a retrospective observational cohort study. *Lancet Infect Dis* 2012;12:696-702.
19. Simpson CR, Lone N, Kavanagh K, et al. Seasonal Influenza Vaccine Effectiveness (SIVE): an observational retrospective cohort study – exploitation of a unique community-based national-linked database to determine the effectiveness of the seasonal trivalent influenza vaccine. *Health Services and Delivery Research* 2013;1(10).
20. Simpson CR, Lone NI, Kavanagh K, et al. Evaluating the effectiveness, impact and safety of live attenuated and seasonal inactivated influenza vaccination: protocol for the Seasonal Influenza Vaccination Effectiveness II (SIVE II) study. *BMJ Open* 2017;7:e014200.
21. Scottish Government. COVID-19 community hubs. Available from: https://www.gov.scot/publications/coronavirus-covid-19-clinical-guidance-care-at-home-housing-support-and-sheltered-housing/pages/covid-19-community-hubs/ (accessed April 3, 2020).
22. National Services Scotland. Information Services Division. SMR Record Type. Available from: https://www.ndc.scot.nhs.uk/Dictionary-A-Z/Definitions/index.asp?Search=S&ID=458&Title=SMR%20Record%20Type (accessed March 24, 2020).
23. National Services Scotland. Information Services Division. SMR01 – General/Acute Inpatient and Day Case. Available from: https://www.ndc.scot.nhs.uk/Dictionary-A-Z/Definitions/index.asp?Search=S&ID=460&Title=SMR01%20-%20General/Acute%20Inpatient%20and%20Day%20Case (accessed March 24, 2020).
24. National Services Scotland. Information Services Division. SMR02 – Maternity Inpatient and Day Care. Available from: https://www.ndc.scot.nhs.uk/Dictionary-A-Z/Definitions/index.asp?Search=S&ID=461&Title=SMR02%20-%20Maternity%20Inpatient%20and%20Day%20Case (accessed March 24, 2020).
25. National Services Scotland. Information Services Division. Deaths. Available from: https://www.isdscotland.org/Health-Topics/Deaths/ (accessed March 24, 2020).
26. National Services Scotland. Information Services Division. Data Quality Assurance. Available from: https://www.isdscotland.org/Products-and-Services/Data-Quality/Assessments/index.asp?Co=Y (accessed March 24, 2020).
27. NHS Scotland. eHealth. HEPMA. Available from: https://www.ehealth.scot/case-studies/hepma/ (accessed March 24, 2020).

For peer review only - http://bmjopen.bmj.com/site/about/guidelines.xhtml

28. Scottish Intensive Care Society Audit Group. Available from: https://www.sicsag.scot.nhs.uk/about/main.html (accessed March 30, 2020).
29. Health Protection Scotland. Data and surveillance. Available from: https://www.hps.scot.nhs.uk/data/ (accessed March 25, 2020).
30. NHS Greater Glasgow and Clyde. West of Scotland Specialist Virology Centre. Available from: https://www.nhsggc.org.uk/about-us/professional-support-sites/laboratory-medicine/laboratory-disciplines/microbiology-and-virology/west-of-scotland-specialist-virology-centre/# (accessed March 25, 2020).
31. Victora CG, Huttly SR, Fuchs SC, Olinto MT. The role of conceptual frameworks in epidemiological analysis: a hierarchical approach. *Int J Epidemiol* 1997; 26(1): 224-7.

For peer review only

Figure 1. Flow diagram for EAVE II project.

ECOSS: Electronic Communication of Surveillance in Scotland; NHS: National Health Service; HEPMA: Hospital Electronic Prescribing and Medicines Administration; RT-PCR: reverse transcriptase polymerase chain reaction; PHS: Public Health Scotland; eDRIS: The electronic Data Research and Innovation Service; ISD: Information Services Scotland.

For peer review only - http://bmjopen.bmj.com/site/about/guidelines.xhtml

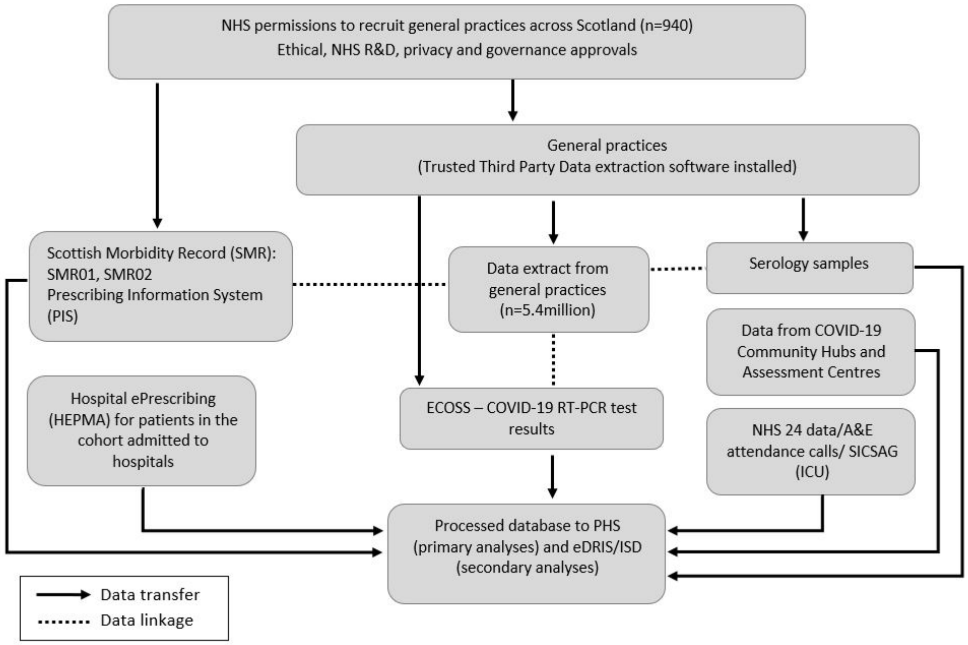

260x171mm (96 x 96 DPI)

Medical Research Council

NIHR | National Institute for Health Research

Prof Aziz Sheikh
University of Edinburgh
Old College
South Bridge
Edinburgh
EH8 9YL

Grant Ref: MC_PC 19075

Date: 7th April 2020

Dear Prof Sheikh

GRANT OFFER: COVID-19 Rapid Response Call 2
GRANT TITLE: COVID-19: Early Assessment of COVID-19 epidemiology and Vaccine/anti-viral Effectiveness (EAVE II)

The MRC is pleased to offer you an award as part of the COVID-19 Rapid Response Call. This Rapid Response is designed to support studies on the COVID-19 virus and epidemic with rapid activation, to enable early and valuable outcomes to be established and/or to access time-dependent resources.

An Award Acceptance Letter is enclosed with this Offer document and must be returned to UKRI within five working days of the date of issue above.

Funding: As a Rapid Response - The MRC confirm the following funding amount, contingent upon the return of the "Award Acceptance Letter". Its funding obligation, and the aggregate amount funded as part of these T&Cs, will be £451,229.00.

Return of the **'Award Acceptance Letter'** will be taken as acceptance of the grant on the terms stated. **The start date of the award can be no later than four weeks from 26/03/2020.** Payment for this grant will be made by invoice quarterly in arrears. Please send your invoice to corporatefinanceextramural@mrc.ukri.org, quoting the purchase order number (PO number to follow). The purchase order number must be quoted on the invoice to ensure payment.

If you have already secured or secure in the future funding for any of the proposed research from another funder then you are required to notify us immediately and we reserve the right to withdraw the offer.

If you are unable to accept the grant, please contact ResearchFundingPolicyandDelivery@mrc.ukri.org quoting the grant reference number and the reason for the decline as soon as possible.

Institutional contribution: This should be consistent with that specified in the original application or as directed in the feedback provided by the MRC.

Remit: Funds should be allocated to the proposal which meet the aims of the COVID-19 Rapid Response Call (as specified at https://mrc.ukri.org/funding/browse/2019-ncov-rapid-response-call/2019-ncov-rapid-response-call/), in a manner consistent with that set out in the original application or as directed in the feedback provided by the MRC.

Reporting: Awardees will be required to complete the MRC's ResearchFish system when notified, the Principal Investigator can add other researchers (who are being funded from the award), to ResearchFish to enable them to complete their own outputs. Given the nature of this call and interest in this study there may be also be ad hoc requests for updates.

For peer review only - http://bmjopen.bmj.com/site/about/guidelines.xhtml

Ethics and related governance: Appropriate ethical and regulatory approvals are required to be in place

For peer review only

before any research requiring such approval is commenced.

Grants are cash limited to the value of the award. RGC 5, 6, of the terms & conditions (below) are superseded by those described in this letter and do not apply.

Post Award

The research organisation is required to record all expenditure on this grant. As part of the reporting, please complete and return via email to corporatefinanceextramural@headoffice.mrc.ac.uk the completed summary of financial expenditure form in ANNEX 1 within 3 months of the end of the award.

Yours sincerely

Dr Joanna Jenkinson
Head of Infections and Immunity
Medical Research Council

For peer review only

Organisation: University of Edinburgh

Grant Holder: Prof Aziz Sheikh

Grant Title: COVID-19: nCoV: Early Assessment of COVID-19 epidemiology and Vaccine/anti-viral Effectiveness (EAVE II)

Starts: 01/04/2020 **Ends: 30/09/2021**

Funds Awarded £451,229.00

Payment Dates: 6 Quarterly payments in arrears (end of June, Sep, Dec, Mar)

In addition to the UKRI standard and MRC additional T&Cs included with this award, the following terms apply:

GRANT CONDITIONS

- The start date of the award can be no later than four weeks of the award notification email.
- Please note that due to the fixed start date, the normal three month start period rules outlined in UKRI Terms and Conditions RGC5 do not apply to this project.
- The investigators must acknowledge the UKRI (MRC), the DHSC (NIHR) support in any publications or events associated with this grant.
- As this is a rapid response, project timeline grant extensions will not be considered.
- In addition to the data sharing terms and conditions at AC10 researchers undertaking work relevant to public health emergencies are required to set in place mechanisms to share quality-assured interim and final data as rapidly and widely as possible, including with public health and research communities and the World Health Organization in accordance with the Joint statement on sharing research data and findings relevant to the novel coronavirus outbreak.
- Please note that this award is currently in confidence pending formal announcement of the funding and should not be shared more widely until the UKRI/DHSC press release has been issued.

Ethical requirements for international grants:

- It is the responsibility of the Principal Investigator and the Research Organisation to ensure that appropriate ethical approval is granted for this study and adhered to, and that no research requiring ethical approval is initiated until it has been granted.
- MRC current policy for research involving humans, http://www.mrc.ac.uk/news-events/publications/research-involving-human-participants-in-developing-societies/ is that for research to be undertaken overseas, both local and UK ethical approval is required.
- For clinical studies involving human participants and/or patients appropriate consent must be obtained.

For peer review only - http://bmjopen.bmj.com/site/about/guidelines.xhtml

- For grants that include the use of animals, the guidance http://www.mrc.ac.uk/news-events/publications/responsibility-in-the-use-of-animals-in-research/ must be adhered to, and in particular: 'When collaborating with other laboratories, or where animal facilities are provided by third parties, researchers and the local ethics committee in the UK should satisfy themselves that welfare standards consistent with the principles of UK legislation (e.g. the ASPA) and set out in this guidance are applied and maintained.'

- The Principal Investigator/ Research Organisation must be prepared to furnish the MRC with a copy of the ethical approval, and any correspondence with the committees, if requested by the Council. The principal investigator must notify the MRC if a regulator or a research ethics committee requires amendments that substantially affect the research question, methodology or costs to the extent that the project is no longer the same as that approved for funding by the MRC.

For peer review only

UK Research and Innovation

UK RESEARCH AND INNOVATION fEC GRANTS STANDARD

TERMS AND CONDITIONS OF GRANT

For peer review only

Contents

Introduction

Application of Standard Terms and Conditions of

Grant Use of Grant Proposal Information

Standard Terms and Conditions of Grant

RGC 1 Variation to Terms and Conditions

RGC 2 Accountability & Responsibilities of the Research

Organisation RGC 3 Research Governance

RGC 3.1 Research Ethics, Misconduct and Conflicts of

Interest RGC 3.2 Use of Animals in Research

RGC 3.3 Health and Safety

RGC 3.4 Equality, Diversity and Inclusion

RGC 3.5 Safeguarding

RGC 3.6 Bullying and

Harassment RGC 3.7

Whistleblowing

RGC 4 Use of Grant

RGC 5 Starting Procedures

RGC 6 Extensions

RGC 7 Monitoring

RGC 7.1 Changes to Project

RGC 7.2 Transfer of a Grant to another Research

Organisation RGC 7.3 Change of Grant Holder

RGC 7.4 Research Monitoring and Evaluation

RGC 7.5 Disclosure and Inspection

RGC 8 Staff

RGC 8.1 Employment

RGC 8.2 Career Development

RGC 8.3 Maternity, Paternity, Adoption and Parental

Leave RGC 8.4 Sick Leave

RGC 9 Equipment

RGC 9.1 Procurement of Equipment

RGC 9.2 Ownership of Equipment

RGC 9.3 Equipment Data

RGC 10 Financial Reporting

RGC 11 Sanctions

RGC 12 Exploitation and Impact

RGC 13 Disclaimer

RGC 14 Status

Annex A:

Definitions

Annex B: Information Sources

Annex C: Version Control

For peer review only

Introduction

UK Research and Innovation fEC Grants Standard Terms and Conditions of Grant

The Standard Terms and Conditions of Grant apply to Research Grants and Fellowships, costed and funded on a Full Economic Costs basis (fEC) and calculated according to the Transparent Approach to Costing (TRAC) or an equivalent methodology, awarded by the following seven UK Research and Innovation (UKRI) Councils:

- Arts and Humanities Research Council (AHRC)
- Biotechnology and Biological Sciences Research Council (BBSRC)
- Economic and Social Research Council (ESRC)
- Engineering and Physical Sciences Research Council (EPSRC)
- Medical Research Council (MRC)
- Natural Environment Research Council (NERC)
- Science and Technology Facilities Council (STFC)

Application of Standard Terms and Conditions of Grant

In these Standard Terms and Conditions of Grant, the words **"We", "Our" or "Us"** refer to **the relevant Council of UKRI awarding the Grant** and **"You" or "Your"** refer to **the Research Organisation in receipt of the Grant**. Other key terms used in these Standard Terms and Conditions of Grant are set out in the Definitions attached at Annex A.

These Standard Terms and Conditions of Grant, together with any applicable Specific Terms and Conditions of Grant required by an individual Council of UKRI comprise the Grant Terms and Conditions on which UKRI awards the Grant to the Research Organisation. Specific Terms and Conditions of Grant will be set out in the Grant Offer Letter.

These Grant Terms and Conditions should be read in conjunction with the sources outlined in Annex B, in the event of any conflict the terms of these Conditions should prevail.

Use of Grant Proposal Information

UK Research and Innovation (UKRI) handles all personal data in accordance with current UK data protection legislation and the EU General Data Protection Regulation (GDPR) where appropriate.

It is the responsibility of the Research Organisation to ensure that both students it funds from UKRI funding and individuals who receive grant funding, or who are later involved in the award, are made aware of how personal data may be used by both UKRI and the Research Organisation. This includes information relating to groups such as students, supervisors, project partners, investigators, named researchers and support staff.

To meet UKRI's obligations for public accountability and the dissemination of information, contents of funded research proposals will also be made available on the Councils' websites and other publicly available sources. As a condition of funding, UKRI may use the data to publish information on awards made. We may also share information with third parties to support, for example, open access publication and reporting outcomes via Researchfish. This includes data submitted through Je-S Student Details (SD).

UKRI is also subject to the UK Freedom of Information Act (2000) and the Environmental Information Regulations (2004) and may be required to release grant information on request, subject to appropriate exemptions.

For peer review only - http://bmjopen.bmj.com/site/about/guidelines.xhtml

Further information is provided by the UKRI Use of grant proposal information addendum

(www.ukri.org/files/funding/tcs/grants-addendum-pdf/) and via the UKRI Privacy Notice (www.ukri.org/privacy-notice/).

Standard Terms and Conditions of Grant

RGC 1 Variation to Terms and Conditions

UKRI reserves the right to amend and vary these Standard Terms and Conditions of Grant and any Specific Terms and Conditions of Grant or applicable policies at any time. The latest version of the Standard Terms and Conditions of Grant apply to all Grants with immediate effect and supersede any previous Standard Terms and Conditions under which a Grant was awarded unless otherwise stated. However, any Specific Terms and Conditions of Grant will still apply. Additional costs incurred as a direct result of changes made to Our Terms and Conditions should be managed within the Grant cash limit. Where the cash limit is exceeded solely due to costs incurred as a result of changes made to Our Terms and Conditions, a case can be made to Us for additional funds on an exceptional basis. The latest version of the Standard Terms and Conditions of Grant are available on the UKRI website at: https://www.ukri.org/funding/information-for-award-holders/grant-terms-and-conditions/

RGC 2 Accountability & Responsibilities of the Research Organisation

RGC 2.1 You are responsible for ensuring that the Project carried out by You, the Grant Holder and any Research Workers or other Third Parties, comply with these Standard Terms and Conditions of Grant and any Specific Terms and Conditions of Grant.

RGC 2.2 You must ensure that the Project is carried out in accordance with all applicable ethical, legal and regulatory requirements including but not limited to relevant provisions of the General Data Protection Regulation, the Data Protection Act 2018, the Bribery Act 2010, the Fraud Act 2006, the Equality Act 2010 and the Modern Slavery Act 2015.

RGC 2.3 You must ensure that Your use of the Grant complies with European Union State Aid[1] law. Where You are informed or You are aware that Your use of the Grant counts as De Minimis Aid[2], the financial limit must not be breached. All other use of the Grant which counts as Aid must fall under the General Block Exemption Regulation[3], it is Your responsibility to inform Us of any State Aid derived throughout the Grant Period. You acknowledge that if You breach State Aid law, UKRI may be required to recover some or all Grant funding, together with interest. For further information please refer to the Department for Business Innovation and Skills: The State Aid Manual.

RGC 2.4 You are accountable for the conduct of the Project including the conduct of the research, the use of public funds and the proper financial management of the Grant in accordance with these Standard Terms and Conditions of Grant and any Specific Terms and Conditions of Grant, whether the Project is carried out by You or the Grant Holder, Research Workers or other Third Party.

RGC 2.5 You must ensure that the Grant is spent in a way that is consistent with the purpose and conditions set out in the Offer Letter.

RGC 2.6 You must carry out appropriate due diligence on any Third Parties used to deliver any part of the Project and shall ensure in particular, that such Third Parties comply with these Standard Terms and Conditions of Grant and any Specific Terms and Conditions of

[1] Including but not limited to Articles 107 to 109 of the Treaty on the Functioning of the European Union, the General Block Exemption Regulation and any Enabling Regulation, as amended from time to time

[2] Commission Regulation (EU) No 1407/2013

[3] Commission Regulation EU No. 651/2014

Grant. At UKRI's request, You must provide details of expenditure of the Grant by any Third Party. Where all, or part, of the Project is carried out by Third Parties based overseas, You must follow the UKRI International Due Diligence Guidance: https://www.ukri.org/files/funding/due-diligence-guidance-for-ukros-pdf/

RGC 2.7 You must ensure that any part of the Full Economic Cost not funded by the Grant is committed to the Project before it starts.

RGC 2.8 You must have adequate business continuity plans in place to ensure minimum operational interruptions to the Project.

RGC 2.9 In order to foster a research culture which values, recognises and supports public engagement, You must adopt the principles, standards and good practice for public engagement with research set out in the 2010 Concordat for Engaging the Public with Research: https://www.ukri.org/public-engagement/research-council-partners-and-public-engagement-with-research/embedding-public-engagement/

RGC 2.10 You must notify UKRI of any changes to Your constitution, legal for, membership structure (if applicable) or ownership, including those that might affect Your eligibility to hold the Grant, or to deliver the Project or any other changes which affect Your ability to comply with the Grant Terms and Conditions.

RGC 2.11 You must ensure that the requirements of the Employing Organisation under the UK Policy Framework for Health and Social Care Research (or equivalent) are met for research involving National Health Service (or equivalent) patients, their organs, tissues or data, and that the necessary arrangements are in place with partner organisations. Where You also accept the responsibilities of a Sponsor (as defined in the Policy Framework), You must also ensure that the requirements for Sponsors are met.

RGC 2.12 Peer review is an integral part of the application process and ensures research of the highest calibre is funded. Investigators and named Researchers on this Grant are expected to make all reasonable efforts to undertake the peer review of proposals for UKRI when invited to do so, unless there is a conflict of interest or the proposal is outside of their area of expertise.

RGC 2.13 By accepting this Grant You are confirming that the Grant Holder has not already received competitively obtained research or support funding from any source, for the same research Project that this Grant has been awarded by Us to support. We reserve the right to terminate the Grant should We find that the Grant Holder has been or is in receipt of the aforementioned duplicate funding, either before or during the Grant Period.

RGC 3 Research Governance

RGC 3.1 Research Ethics, Misconduct and Conflicts of Interest

RGC 3.1.1 You are responsible for ensuring that ethical issues relating to the Project are

For peer review only - http://bmjopen.bmj.com/site/about/guidelines.xhtml

identified and brought to the attention of the relevant approval or regulatory body. Before any such work requiring approval begins, approval must have been granted by the relevant body.

RGC 3.1.2 You must follow Our Policy and Guidelines on Governance of Good Research Conduct at: https://www.ukri.org/about-us/policies-and-standards/research-integrity/ and ensure that the requirements set out in the Concordat to Support Research Integrity (2012) are met. In particular, You are responsible for ensuring all necessary permissions are obtained before the Project begins, that there is clarity in roles and responsibility among Grant Holders, Research Workers, and Third Parties, as well as investigating and reporting unacceptable research conduct. Any potential conflicts of interest in research identified at the point of application must be declared to Us and subsequently managed.

RGC 3.2 Use of Animals in Research

You must comply with the provisions of the Animals (Scientific Procedures) Act 1986, and any amendments, where applicable and ensure that all necessary licences are in place before any work requiring approval takes place. You should also follow the guidance set out in "Responsibility in the use of animals in bioscience research": https://www.nc3rs.org.uk/responsibility-use-animals-bioscience-research

RGC 3.3 Health and Safety

You are responsible for ensuring a safe working environment for all individuals associated with the Project, both on and off-site, and for meeting all regulatory and legislative health and safety requirements.

We reserve the right to require You to undertake a safety risk assessment in individual cases where health and safety may be an issue, and to monitor and audit the actual arrangements made. In the event of a serious incident (e.g. death) we require that you inform us for risk purposes.

RGC 3.4 Equality, Diversity and Inclusion

You are expected to ensure that equality, diversity and inclusion is considered and supported at all stages throughout the performance of the Project, in alignment with Our policies and principles at: https://www.ukri.org/about-us/policies-and-standards/equality- diversity-and-inclusion/ for equality, diversity and inclusion. Your approach to supporting equality, diversity and inclusion is expected to exceed all relevant legal obligations, including but not limited those of the Equality Act 2010.

RGC 3.5 Safeguarding

All relevant safeguarding legislation must be adhered to, We particularly draw your attention to child protection legislation and the Modern Slavery Act 2015. You must have sufficient policies and/or processes in place in order to foster Safeguarding.

RGC 3.6 Bullying and Harassment

You must have clear, well-publicised policies, processes and training in place consistent with good practice as recommended by the Advisory, Conciliation and Arbitration Service's (ACAS) 'Bullying and Harassment in the Workplace: A Guide for Managers and Employers'.

RGC 3.7 Whistleblowing

You must have clear, well-publicised policies and processes in place consistent with good practice recommended by the National Audit Office Assessment Criteria for Whistleblowing policies.

RGC 4 Use of Grant

RGC 4.1 We reserve the right to vary the value of the Grant during its lifetime in accordance with the GDP Deflators published by HM Government or to take into account any other Government decisions affecting the funding available to UKRI.

RGC 4.2 With the exception of RGC 4.3, Directly Incurred and Exceptions funds must not be used to meet the costs of an activity that will fall outside the Grant Period.

RGC 4.3 Expenditure may be incurred prior to the start of the Grant and be subsequently charged to the Grant, provided that it does not precede the date of the Offer Letter.

RGC 4.4 Transfers of funds between fund headings are permitted only within and between Directly Incurred and Exceptions costs, excluding equipment, at the rate applicable for the heading as set out in the award letter. Funds may only be transferred into studentship stipend or fees to supplement an existing studentship post on the Grant. You must not transfer funds to create new posts without prior approval from UKRI. Directly Incurred and Exceptions funds must not be used to meet costs on any other Grant or activity.

Funds can only be transferred and used to meet the cost of activity or activities that meet the agreed aims and objectives of the project. While approval does not need to be sought from Us for transfer of funds (excluding the creation of new posts), We reserve the right to query any expenditure outlined in the Final Expenditure Statement which has not been incurred in line with the Standard Terms and Conditions of Grant and any Specific Terms and Conditions of Grant.

RGC 4.5 Costs associated to Students must not be charged to the Grant. These costs must be met by other resources held by You, which can include UKRI Training Grants if the student holds a UKRI studentship. Students are able to undertake paid work within the institution as casual assistance, this should be evidenced with a clear audit trail and should not form part of the formal studentship training.

RGC 5 Starting Procedures

RGC 5.1 You must formally accept the Grant by completing and returning the Offer Acceptance within 10 working days of the issue of the Offer Letter.

RGC 5.2 You must submit the Start Confirmation within 42 (calendar) days of the Project starting. The date entered on the Start Confirmation will be the Official Start Date of the Grant. The Official Start Date may be delayed by up to 3 months from the start date shown in the Offer Letter, but the duration of the Grant will remain unchanged. The Grant may lapse if the Project is not started within 3 months of the start date in the Offer Letter. The start of the Grant may precede the start date shown in the Offer Letter, but must not be earlier than the issue date of the Offer Letter itself.

RGC 6 Extensions

RGC 6.1 The duration of the Grant ("Grant Period") may be extended after the Official Start Date by up to 12 months without additional funding subject to Our prior written approval. For further information, see the UKRI fEC Grant Guidance document.

For peer review only - http://bmjopen.bmj.com/site/about/guidelines.xhtml

RGC 6.2 For Fellowship Grants, the Grant Period may also be extended to cover familial leave,

extended jury service or paid sick leave after the Official Start Date for a period in line with the Terms and Conditions of the Fellow's employment. For further information, see the UKRI fEC Grant Guidance document.

RGC 7 Monitoring

RGC 7.1 Changes to Project

You must inform and consult Us if there are any significant changes that may affect the progress, delivery or State Aid status of the Project. No substantive changes to the experimental design of a project involving the use of animals or human participation, which might affect the ethical characteristics of the award, are permitted without the prior approval of UKRI.

If You propose to make significant changes to the Project, UKRI may require revised proposals for its approval and reserves the right to make a new Grant in place of the existing Grant, or to revise, retain or terminate the existing Grant.

RGC 7.2 Transfer of a Grant to another Research Organisation

RGC 7.2.1 The Grant may be transferred to another eligible organisation, providing that it can provide a suitable environment to enable the project to be successfully completed; this will be subject to prior written approval of UKRI. Written agreement to this is required from both the relinquishing and receiving organisations.

RGC 7.2.2 Grant funding will not be revised following transfer. The receiving organisation must confirm that it will provide any additional resources needed to complete the project by returning an Offer Acceptance.

RGC 7.3 Change of Grant Holder

RGC 7.3.1 For Research Grants, You must submit any proposed changes of Grant Holder to UKRI for approval via the Grant Maintenance facility in Je-S.

RGC 7.3.2 For Fellowship Grants, changes to the Grant Holder are not permitted. In the event of the research fellow's resignation or other termination of their employment, the Grant will terminate automatically.

RGC 7.4 Research Monitoring and Evaluation

RGC 7.4.1 You must use Our nominated online system to submit information for monitoring and evaluation purposes on the outputs and outcomes and impacts of the Project during and for some years after the expiry of the Grant Period. Further information on reporting requirements can be found on the UKRI website: https://www.ukri.org/funding/information-for-award-holders/research-outcomes/help-and-guidance/. Failure to comply with the reporting requirements will result in suspension of Grant payments and no further proposals will be considered by UKRI where the Grant Holder is named as the Principal or Co-Investigator.

RGC 7.4.2 Exceptionally We may require a separate End of Award Report on the conduct and outcome of the Project. If required You must submit the report within 3 months of the end of the Grant Period. No further application from a Grant Holder will be considered while an End of Award Report is overdue.

RGC 7.4.3 We reserve the right to call for periodic updates on the Project's progress or to visit

For peer review only

the Project team, or request participation in evaluation studies. The Grant Holder must make all reasonable efforts, if so invited, to respond to requests for information or to attend events or activities organised by UKRI concerning the research undertaken, including requests or events after the end of the Grant Period.

RGC 7.5 Disclosure and Inspection

RGC 7.5.1 We shall be entitled to inspect any financial or other records and procedures associated with the Grant as are reasonably required to verify the regularity and propriety of Grant expenditure, or to appoint any other body or individual for the purpose of such inspection. This includes expenditure by Third Parties.

RGC 7.5.2 If We request it, You must provide a statement of account for the Grant, independently examined by an auditor who is a member of a recognised professional body, certifying that the expenditure has been incurred in accordance with the Grant Terms and Conditions.

RGC 7.5.3 You must report to us any investigations and their outcomes into research misconduct associated with the Grant in advance of any enquiry whether informal or formal, and upon request, provide information on Your management of research integrity and ethics as described at: www.ukri.org/about-us/policies-and-standards/research-integrity/. In addition, You must provide details of any retractions or withdrawal of submissions/publications, any allegations, proven or not, of cases of fraud and any other complaint or investigation into dishonesty, fraudulent activities or business misconduct, by any regulatory body or the police into Your activities or those of Your staff.

RGC 7.5.4 We will undertake periodic reviews of Research Organisations within the Funding Assurance Programme to seek assurance that Grants are managed in accordance with the Terms and Conditions under which they are awarded.

RGC 8 Staff

RGC 8.1 Employment

You are wholly responsible for staff funded from the Grant, including Research Fellows, and accept all duties owed to and responsibilities for these staff, including, without limitation, their terms and conditions of employment, and their training and supervision, arising from the employer/employee relationship. You must appoint a Research Fellow as an employee for the full duration of the award.

RGC 8.2 Career Development

You are expected to adopt the principles, standards and good practice for the management of research staff set out in the 2019 Concordat to Support the Career Development of Researchers, including any subsequent amendments.

RGC 8.3 Maternity, Paternity, Adoption and Parental Leave

RGC 8.3.1 At the end of the Grant Period We will reimburse costs incurred by You to cover any additional net parental leave costs that cannot be met within the announced grant cash limit including Statutory Maternity, Paternity and Adoption Pay for staff, within the Directly Incurred and Exceptions fund headings. This will be payable only for the percentage of time

For peer review only

that the staff are contracted on the Grant.

RGC 8.3.2 Within the announced grant cash limit, the Grant may be used to meet the costs of making a substitute appointment and/or extending the Grant to cover a period of parental leave for staff within the Directly Incurred and Exceptions fund headings (as outlined above). Directly Allocated and Indirect funds will not be increased as a result of such extensions.

RGC 8.3.3 You will be responsible for any liability for parental leave pay for staff supported by the Grant outside the original Grant Period.

RGC 8.3.4 Fellows are entitled to take parental leave in accordance with the terms and conditions of their employment. We will consider requests for a Fellowship Grant to be placed in abeyance during the absence of the Research Fellow for parental leave, and the period of the Fellowship extended by the period of leave. We will also consider requests to continue the Fellowship on a flexible or part-time basis to allow the Research Fellow to meet caring responsibilities.

RGC 8.4 Sick Leave

RGC 8.4.1 At the end of the Grant Period, We will reimburse You for any additional net sick leave costs that cannot be met within the announced Grant cash limit for staff within the Directly Incurred and Exceptions fund headings, except where You have already recovered these costs by claiming Statutory Sick Pay from HMRC. This will be payable only for the percentage of time that the staff are contracted on the Grant.

RGC 8.4.2 Within the announced grant cash limit, the Grant may be used to meet the costs of making a substitute appointment and/or extending the Grant to cover a period of sick leave for staff within the Directly Incurred and Exceptions fund headings (as outlined RGC 8.4.1). Directly Allocated and Indirect funds will not be increased as a result of such extensions.

RGC 8.4.3 You will be responsible for any liability for sick leave pay for staff supported by the Grant outside the original Grant Period.

RGC 8.4.4 Where there is a continuous period of sick leave in excess of 3 months, You may request approval for a substitute appointment to safeguard progress on the Project. Where a Research Assistant has been on sick leave in excess of 3 months, You must comply with all obligations to consider reasonable adjustments before making a substitute appointment. Where a Research Assistant has been on sick leave for an aggregate (not necessarily continuous) period in excess of 3 months, where this is due to a single condition or a series of related conditions, You may request an extension to the duration of the project.

RGC 8.4.5 Fellowship Grants: Fellows are entitled to take sick leave in accordance with the Research Organisation's terms and conditions. If requested, consideration will be given to allowing a fellowship grant to be placed in abeyance during the absence of the Research Fellow due to sick leave, and the period of the fellowship extended by the period of sick leave. The additional salary costs for the fellow (pro rata to their percentage FTE on the fellowship) should be claimed, as necessary, at the end of the extended period

For peer review only

RGC 9 Equipment

RGC 9.1 Procurement of Equipment

The procurement of equipment, consumables and services, including maintenance, must comply with all relevant national and EU legislation and consideration must be given to the energy and waste implications of all procurements. For contracts over £25,000, excluding VAT, professionally qualified procurement staff must be consulted before the procurement process begins. Any proposal to purchase equipment in the last 6 months of the Grant must be pre-approved by UKRI.

RGC 9.2 Ownership of Equipment

You must inform us if the need for the equipment diminishes substantially or it is not used for the purpose for which it was funded during the Grant Period. We reserve the right to determine the disposal of such equipment and to claim the proceeds of any sale. Any proposal to transfer ownership of the equipment during the period of the Grant requires the prior approval by UKRI.

RGC 9.3 Equipment Data

All new equipment purchased over £138,000 (£115,000 ex VAT) must be registered on the "Equipment.data" national database.

RGC 10 Financial Reporting

RGC 10.1 You are accountable for funds dispersed and are responsible for the timely and accurate submission of all expenditure reports required under the Terms and Conditions of Grant, including the submission of an expenditure statement within 3 months of the end of the Grant Period. We are entitled to require You to provide supplementary information in support of an interim or final expenditure statement. Once an expenditure statement has been received and the expenditure incurred has been reconciled against payments made, it will be considered as final. Any unspent funds will be recovered.

RGC 10.2 You must retain all accounting information relating to the Grant for the current financial year plus the subsequent six years after the submission date of the final expenditure statement.

RGC 10.3 If We send an Annual Statement to return showing payments made by UKRI during the previous financial year for all the Grants You hold, You must complete and return the statement by the specified deadline.

RGC 11 Sanctions

RGC 11.1 We reserve the right to impose financial sanctions and/or additional measures if You do not comply with Your obligations as set out in these Standard Terms and Conditions of Grant and any Specific Terms and Conditions of Grant.

RGC 11.2 If the End of Award Report (if required) or the Financial Expenditure Statement is not received within 3 months of the end of the Grant Period, UKRI will recover 20% of expenditure incurred on the Grant. All payments will be recovered if the report or statement is not received within 6 months of the end of the Grant. You may appeal against a sanction, but must do so within 60 days of the pay run in which the sanction was imposed.

For peer review only

RGC 11.3 Where an Organisation is deemed to be non-compliant in the application of TRAC, a

minimum sanction of 75% of the non-compliant rate may be applied, where an Organisation is applying rates which are materially inaccurate (>10% variance on any single rate). These sanctions would only apply to future applications, until a time that UKRI Funding Assurance are satisfied that remedial measures are implemented.

RGC 12 Exploitation and Impact

RGC 12.1 Unless otherwise agreed, all intellectual property shall belong to the party that generates them. Where the Grant is associated with more than one Research Organisation and/or other project partners, the basis of collaboration between the organisations including ownership of intellectual property and rights to exploitation, is expected to be set out in a formal collaboration agreement.

RGC 12.2 You are responsible for ensuring that all parties engaged in the research make every reasonable effort to ensure that the intellectual assets obtained in the course of the research, whether protected by intellectual property rights or not, are used to the benefit of society and the economy.

RGC 12.3 In individual cases, We reserve the right to retain ownership of intellectual assets, including intellectual property (or assign it to a third party under an exploitation agreement) and to arrange for it to be exploited for the national benefit and that of the Research Organisation involved.

RGC 12.4 The Grant Holder shall, subject to the procedures laid down by the Research Organisation, publish the results of the research funded by the Grant in accordance with normal academic practice and Our policy on Open Access: https://www.ukri.org/files/legacy/documents/rcukopenaccesspolicy-pdf/. Other forms of media communication, including media appearances, press releases and conferences, must acknowledge the support received from Us, quoting the Grant reference number if appropriate.

RGC 13 Disclaimer

RGC 13.1 UK Research and Innovation accepts no liability, financial or otherwise, for expenditure or liability arising from the research funded by the Grant except as set out in these Terms and Conditions, or otherwise agreed in writing.

RGC 13.2 UKRI reserves the right to amend the payment profile at its discretion. You will be advised, in advance, of any such change. Changes to payment profiles may affect the overall value of the Grant.

RGC 13.3 UKRI reserves the right to terminate the Grant at any time, subject to reasonable notice and to any payment that We agree may be necessary to cover outstanding and unavoidable commitments. If a Grant is terminated or reduced in value, no liability for payment, redundancy or any other compensatory payment for the dismissal of staff funded by the Grant will be accepted, but, subject to the provisions of RGC 10 Financial Reporting, negotiations will be held with regard to other contractual commitments and concerning the disposal of assets acquired under the research grant.

RGC 13.4 Where studies are carried out in an NHS Trust or equivalent, the Trust or equivalent has a duty of care to its patients. UK Research and Innovation does not accept liability for any failure in the Trust's duty of care, or any negligence on the part of its employees.

For peer review only - http://bmjopen.bmj.com/site/about/guidelines.xhtml

RGC 14 Status

RGC 14.1 The Terms and Conditions of Grant which include these Standard Terms and Conditions of Grant and the Specific Terms and Conditions of Grant will be governed by the laws of England and Wales and all matters relating to the Terms and Conditions will be subject to the exclusive jurisdiction of the courts of England and Wales.

RGC 14.2 If any provision of these Terms and Conditions is found by a court or other legitimate body to be illegal, invalid or unreasonable, it will not affect the remaining Terms and Conditions which will continue in force.

RGC 14.3 The Terms and Conditions of Grant contain the whole agreement between UKRI and the Research Organisation in relation to the Grant and neither party intends that any of these Terms and Conditions should be enforceable by any third party.

For peer review only

Annex A

Definitions

Co-Investigator: A person who assists the Grant Holder in the management and leadership of the Project.

Council: Any of the bodies listed under the Introduction.

Directly Allocated Costs: Costs of resources used by the Project that are shared by other activities. They are charged on the basis of estimates rather than actual costs and do not represent actual costs on a project by project basis.

Directly Incurred Costs: Costs that are explicitly identifiable as arising from the conduct of the Project which are charged as the cash value actually spent and are supported by an audit record.

End of Award Report: A report which the Grant Holder must provide at the end of the Grant Period, detailing the outputs, outcomes and impacts of the project to date.

Exceptions: Directly Incurred Costs that Councils fund at 100% of fEC subject to actual expenditure incurred, or items that are outside fEC.

Fellowship Grant: An award made through a fellowship competition providing a contribution to the support of a named individual. It covers the cost of the time dedicated by the fellow to their personal research programme, and may or may not include research support costs.

Full Economic Costs (fEC): A cost which, if recovered across an organisation's full programme, would recover the total cost (direct, indirect and total overhead) including an adequate recurring investment in the organisation's infrastructure.

Funding Assurance Programme: A programme of visits and office based tests by UKRI to seek assurance that grant funds are used for the purpose for which they are given and that grants are managed in accordance with the terms and conditions under which they are awarded Gran Support for a proportion of the full economic costs of the Project. A Grant may be either a Research Grant or a Fellowship.

Grant Holder: The person to whom the Grant is assigned and who has responsibility for the intellectual leadership of the Project and for the overall management of the research funded by the Grant. The Grant Holder is either the Principal Investigator (in the case of a Research Grant) or a Research Fellow (in the case of a Fellowship Grant).

Grant Period: The duration of time between the Project start and end date.

Grant Terms and Conditions: The Standard Terms and Conditions of Grant together with the Specific Terms and Conditions of Grant that together comprise the basis on which the Grant is awarded to the Research Organisation.

Indirect Costs: Non-specific costs charged across all projects based on estimates that are not otherwise included as Directly Allocated Costs. They include the costs of the Research Organisation's administration such as personnel, finance, IT, legal, general laboratory, office consumables, library and some departmental services.

Je-S: Joint Electronic Submissions system used for the submission of Grant related information.

Offer Acceptance: A document to be completed and returned by the Research Organisation either accepting or declining the Grant.

Grant Offer Letter / Offer Letter: An official document setting out specific details of the Grant, including the Project start and end date, Grant value and any Specific Terms and Conditions of the Grant as required by the relevant Council.

Official Start Date: The official start date of the Grant, as set out in the Start Confirmation.

Project: The project funded by the Grant as set out in the Offer Letter.

Research Grant: A contribution to the costs of the research Project which has been assessed as eligible for funding through the procedures established by the relevant Council.

Research Organisation (RO)/Grant Awardee: The organisation to which the Grant is awarded and which takes responsibility for the management of the Project and accountability for funds provided.

Research Worker: Any person or third party working in any capacity on the Project.

Specific Terms and Conditions of Grant/Specific Conditions: The specific conditions of grant required in addition to the Standard Terms and Conditions on a Grant by an individual Council of UKRI.

Standard Conditions of Grant/Standard Conditions: The Standard Terms and Conditions of Grant published on UKRI's website at: www.ukri.org/funding/information-for-award-holders/grant-terms-and-conditions/

Start Confirmation: Confirmation of the date on which the Project commences, as notified by the Research Organisation to UKRI.

Studentship: The term used for the funding award made by a Research Organisation to a student for the purpose of undertaking postgraduate training leading to the award of a

For peer review only

postgraduate degree.

Third Party: Any person/organisation to which the award holding RO passes on any of the Grant funds awarded by the Council.

Transparent Approach to Costing (TRAC): An agreed methodology used by universities and other higher education bodies for calculating full economic costs.

For peer review only

Annex B

Information Sources

These Grant Terms and Conditions should be read in conjunction with the following sources. In the event of any conflict the terms of these Conditions should prevail:

1) UKRI Use of grant proposal information addendum: www.ukri.org/files/funding/tcs/grants-addendum-pdf/
2) UKRI Privacy Notice:www.ukri.org/privacy-notice/
3) UKRI Grant Terms and Conditions web page: www.ukri.org/funding/information-for-award-holders/grant-terms-and-conditions/
4) State Aid: Including but not limited to Articles 107 to 109 of the Treaty on the Functioning of the European Union, the General Block Exemption Regulation and any Enabling Regulation, as amended from time to time
5) De Minimis Aid: Commission Regulation (EU) No 1407/2013
6) General Block Exemption Regulation: Commission Regulation EU No. 651/2014
7) Department for Business Innovation and Skills: The State Aid Manual
8) UKRI International Due Diligence Guidance: www.ukri.org/files/funding/due-diligence-guidance-for-ukros-pdf/
9) Concordat for Engaging the Public with Research: www.ukri.org/public-engagement/research-council-partners-and-public-engagement-with-research/embedding-public-engagement/
10) UK Policy Framework for Health and Social Care Research
11) Policy and Guidelines on Governance of Good Research Conduct: www.ukri.org/about-us/policies-and-standards/research-integrity/
12) Concordat to Support Research Integrity (2012)
13) Animals (Scientific Procedures) Act 1986
14) Responsibility in the use of animals in bioscience research guidance: https://www.nc3rs.org.uk/responsibility-use-animals-bioscience-research
15) UKRI Policies and Principles for Equality, Diversity and Inclusion: www.ukri.org/about-us/policies-and-standards/equality-diversity-and-inclusion/
16) Equality Act 2010
17) Modern Slavery Act 2015
18) Advisory, Conciliation and Arbitration Service (ACAS) 'Bullying and Harassment in the Workplace: A Guide for Managers and Employers'
19) UKRI FEC Grant Guidance: https://www.ukri.org/funding/information-for-award-holders/grant-terms-and-conditions/
20) Research Outcome Reporting Requirements: www.ukri.org/funding/information-for-award-holders/research-outcomes1/help-and-guidance/
21) Research Integrity: www.ukri.org/about-us/policies-and-standards/research-integrity/
22) 2019 Concordat to Support the Career Development of Researchers
23) Open Access Policy: www.ukri.org/files/legacy/documents/rcukopenaccesspolicy-pdf/

For peer review only

Annex C

Version Control

Version	Date Implemented	Changes
1.0	13 May 2016	1. Format changes<br>2. Grammar and spelling changes<br>3. All equipment specific terms brought together under new RGC11<br>4. Renumbering<br>5. New Conditions added;<br>• RGC 11.5 Equipment Data<br>• RGC 18 Contact Sanctions<br>5. Change to 'RGC 6 Transfers of Funds between Fund Headings' to include sentence regarding associated students.<br>6. Adding of version control (website version only)
1.1		1. Addition of assurance statements and compliance with grant standards
2.0	01 August 2017	Updated due to RCUK Funding Assurance requirements:<br><br>• Clarifying responsibility for cascading T&Cs to third parties including due diligence checks and monitoring of compliance.<br>• Reinforcing accountability for use of public funds and proper financial management. Implication of State Aid laws.<br>• Clarification of expected retention times for research/training grant documentation.<br>• Guidance on ensuring value for money.<br>• Expanding the "Inspection" section to cover "Disclosure" to clarify research council requirements and expectations in this area.<br>• The ability for Research Councils to impose "additional measures" where non-compliance of T&Cs is identified.
3.0	12 January 2018	Updated to include:<br><br>• Addition in "Definitions" under Data Protection Regulations section, to include GDPR<br>• New condition RGC 2.6 Modern Slavery Act 2015<br>• Expanding RGC 8 Staff to include NHS clinical sessions in approved tasks which may be undertaken<br>• Update to 11.5 Equipment Data to include recent changes to OJEU levels.<br>• New condition RGC 25 Transfer to UK Research & Innovation
3.1	23 March 2018	RCG 11.5 Equipment Data changed to restore previous limits as RCUK no longer links to the Government OJEU limits.
3.2	03 April 2018	Links updated to UKRI site
4.0	28 June 2019	Terms and Conditions reviewed, updates include:<br><br>1. Renumbering of conditions

For peer review only

		2. New conditions added:<br>• RGC 2.12 Peer Review<br>• RGC 2.13 Duplicate Funding<br>• RGC 3.4 Equality, Diversity and Inclusion<br>• RGC 3.5 Safeguarding<br>• RGC 3.6 Bullying and Harassment<br><br>3. Conditions removed:<br>• RGC 11.2 Ownership of Equipment<br>• RGC 25 Transfer to UK Research & Innovation<br><br>4. Conditions updated:<br>• RGC 8.3.1 Maternity, Paternity, Adoption and Parental Leave<br>• RGC 8.4.1 Sick Leave<br>• RGC 4.5 Use of Grant<br>• RGC 2.10 and RGC 7.5.3 have been updated to comply with the new Standard 7 of the Government Functional Standard for General Grants.
5.0	06 March 2020	Terms and conditions reviewed, updates include:<br><br>1. New conditions added:<br>• RGC 3.7 Whistleblowing<br><br>2. Conditions updated:<br>• RGC 1 Variation to Terms and Conditions<br>• RGC 2.3 State Aid<br>• RGC 7.1 Changes to Project – State Aid<br>• RGC 8.3.1 Maternity, Paternity, Adoption and Parental Leave<br>• RGC 8.4.1 Sick Leave<br>• RGC 9.1 Procurement of Equipment

For peer review only

MRC Additional Terms and Conditions

The MRC additional terms and conditions of funding supplement those of UKRI. These conditions set out

operational, legislative and ethical requirements relating to medical research. The MRC reserves the right to vary these additional terms and conditions.

Research organisations and award holders* have absolute responsibility for ensuring all required licenses, approvals, permissions and consent are in place before any research is undertaken and that these are followed.

*Award Holders are all MRC Grant Holders and recipients of MRC Unit and Institute funding (programme leaders). MRC reserves the right to audit at any time without prior notice:
-That required licenses, approvals, permissions and consent are in place, or were in place when the activity occurred.

-Compliance with the terms and conditions set out here

AC1 Responsibilities of the Research Organisation: Clinicians

The research organisation is responsible for ensuring all clinicians
supported by MRC funding are aware they are individually responsible for maintaining appropriate professional indemnity insurance. This should be with a professional defence organisation for any activities not covered by NHS indemnity arrangements or by additional provision made by the research organisation. MRC will not meet the costs of such cover.

The research organisation is responsible for ensuring any honorary clinical contracts required by clinical staff have been obtained prior to the start of the research.

The MRC expects the research organisations to abide by the 'UK clinical academic training in medicine and dentistry: principles and obligations' (https://mrc.ukri.org/news/browse/improving-support-for-clinical-academics/).

AC2 Clinical Responsibilities

Clinical award holders (Clinical Research Training Fellowships, Clinician Scientist Awards, Senior Clinical Fellowships or Clinical Academic Research Partnerships) may not work more than the time commitment for clinical duties stated in their proposal. For the majority, this will equate to up to 20% (on average over the lifetime of the grant) of their normal working hours, which they may choose to spend on NHS clinical sessions, teaching and demonstrating, or research activities beyond the scope of their fellowship. Exceptions are made for surgeons and fellows undertaking patient-oriented research, who may undertake up to 40% of their time on these duties. This is not in addition to the six hours per week all research staff supported full-time by an MRC grant or fellowship may undertake under RGC 8 of the UKRI Terms and Conditions of Research Council fEC Grants (https://www.ukri. org/funding/information-for-award-holders/grant-terms-and-conditions/).

AC3 Mouse Strains

MRC supports a central repository of mouse strains - the MRC mouse Frozen Embryo and Sperm Archive (FESA) at Harwell. Award holders are expected to contact FESA to highlight mouse strains engineered, or characterised using MRC funds, and are encouraged to deposit these strains with the archive.

Depositors retain ownership of strains and there is currently no charge for depositing strains to make them freely available to the academic community.

FESA aims to ensure that valuable mouse strains are safeguarded, that the need to maintain colonies of live mice for long periods of time is reduced, and that the significant investment in engineering strains is capitalised upon fully. MRC award holders planning mouse research should contact FESA at the earliest opportunity.

For help with the requirements of AC5-AC13 please contact MRC Regulatory Support Centre: (https://mrc.ukri. org/research/facilities-and-resources-for-researchers/regulatory-support-centre/).

AC4 Human Participants in Research

MRC expects all research involving human participants to be undertaken in accordance with its policies and guidance available from https://mrc.ukri.org/research/policies-and-guidance-for-researchers/#ethics. These include:

-Good Research Practice (2012);

Using information about people in health research (2016-17)

For peer review only

-Human Tissue and Biological Samples for Use in Research (2014);
-Medical research involving adults who cannot consent (2007);
-Medical Research Involving Children (2004);
-Guidelines for the management of global health trials (2017).

Research organisations and award holders have absolute responsibility for ensuring that investigations being undertaken within NHS premises, nursing or residential homes or NHS service establishments, schools, or any other organisations, do not take place without the explicit approval of the appropriate authority in advance.

AC5 Approvals

Independent Research Ethics Committee approval is required for research that involves human participants (whether patients or healthy volunteers) or records. In the case of research involving NHS patients, premises or records, this will be an NHS Research Ethics Committee (REC). Such approval is also required for certain studies of human tissues. Further guidance on when NHS REC approval is required can be found at (http://www.hra-decisiontools.org.uk/ethics/).

In the case of social science research, the MRC recommends that award holders follow the ESRC Framework for Research Ethics (revised 2015, https://esrc.ukri.org/funding/guidance-for-applicants/research-ethics/), which highlights the responsibility of the research organisation for ensuring that the research is subject to appropriate ethics review. In some case's this review is required by an NHS REC, for further guidance please see (https://www.hra.nhs.uk/approvals-amendments/what-approvals-do-i- need/research-ethics-committee-review/).

MRC does not need to be routinely notified by the award holder of amendments required by a regulator or a REC unless they relate to urgent safety measures and/or substantially change the research approved for funding by the MRC.

AC6 Payments and incentives in research

Payments to healthy volunteers participating in clinical research are allowable, provided that the payment is for expense, time and inconvenience and is not at a level which would induce people to take part in studies against their better judgement. Further guidance on payments and incentives in research can be found at https://www.hra.nhs.uk/about-us/committees-and- services/nreap/.

AC7 Clinical Trials

When research involves MRC-funded clinical trials, award holders must act in accordance with MRC policy on UK clinical trials regulations (https://mrc.ukri.org/research/policies-and-guidance-for-researchers/clinical-research-governance/clinical-trials- regulations/), in relation to ethical, sponsorship, reporting, monitoring and publication requirements. Research involving trial oversight and management for MRC-funded clinical trials conducted in lower and middle income countries (LMICs), should refer to the MRC guidelines for management of global health trials https://mrc.ukri.org/funding/science-areas/global-health-and- international-partnerships/funding-partnerships/joint-global-health-trials/

-An independent Trial Steering Committee, and in most cases a Data Monitoring Committee, must be set up to oversee the conduct of the trial, with an MRC representative acting as an observer. In exceptional circumstances for particularly low risk trials, a researcher may seek approval from the MRC Programme Manager for more limited TSC and/or DMC oversight structures.

-MRC-funded trials must be registered with an International Standardised Randomised Control Trial Number (ISRCTN) on the ISRCTN Registry (http://www.isrctn.com/). The unique identification number must be used in publications and provided to MRC by adding it to Researchfish within a year of the trial starting. Failure to provide this number will result in suspension of funding.

-Results of MRC-funded trials (whether positive or negative) must be published without unreasonable delay and within 24 months of completion of the study. Results should be reported in accordance with the recommendations in the CONSORT statement (http:
//www.consort-statement.org/). Before results are published, they must be discussed by the Trial Steering Committee.

-Any contribution to an MRC-funded trial by another body, such as a pharmaceutical company (donation of drugs etc.), must be the subject of a collaboration agreement between the parties (see AC20).

For peer review only - http://bmjopen.bmj.com/site/about/guidelines.xhtml

AC8 UK Policy Framework for Health and Social Care Research

Research involving NHS (or HSC in Northern Ireland) patients, their organs, tissues or data which falls within the scope of the UK Policy Framework for Health and Social Care Research (https://www.hra.nhs.uk/planning-and-improving-research/policies- standards-legislation/uk-policy-framework-health-social-care-research/) must comply with MRC policy on the health departments' research governance framework (https://mrc.ukri.org/research/policies-and-guidance-for-researchers/clinical-research- governance/health-departments-research-governance/).
MRC requires research organisations to ensure sponsorship responsibilities are clearly identified, the research undertaken complies with the requirements of the employing organisation set out in the UK policy framework, and that agreements and systems are in place with NHS Trusts and other partner organisations, including commercial organisations, to comply with the framework. Systematic documentation of key decisions and approvals, particularly in relation to work with patients, their organs, tissues and data is crucial.

AC9 Medical Records

When research involves the use of medical records, the award holder must act in accordance with the principles set out in data protection legislation (https://ico.org.uk/for-organisations/guide-to-data-protection/introduction-to-data-protection/some-basic- concepts/) and the NHS requirements to protect patient confidentiality. Advice on these requirements is available from the MRC Regulatory Support Centre.

All researchers handling personal data must have clearly established obligations to maintain confidentiality (e.g. formalised within policy written by their research organisations or through professional codes of conduct).

Research involving identifiable patient-level data will require NHS Research Ethics Committee approval and may also require additional approvals. In England and Wales research involving identifiable patient-level data, without patient consent, is covered by "Section 251" of The National Health Service Act 2006 and requires additional approval via the Health Research Authority's Confidentiality Advisory Group (https://www.hra.nhs.uk/approvals-amendments/what-approvals-do-i-need/confidentiality-advisory- group/). In Scotland, decisions on disclosure of identifiable patient-level data are made by Caldicott Guardians (see https://www. informationgovernance.scot.nhs.uk/pbpphsc/ for further details).

AC10 Data Sharing

Award holders must comply with the MRC policy on research data sharing (https://mrc.ukri.org/documents/pdf/mrc-data-sharing- policy/ along with the MRC policy on sharing of research data from population and patient studies (https://mrc.ukri. org/research/initiatives/health-and-biomedical-informatics/access-governance-and-ethics/).

When research involves clinical trials, clinical intervention studies, public health intervention studies or observational studies award holders must comply with the MRC policy on open research data from clinical trials and public health interventions (https://mrc.ukri. org/research/policies-and-guidance-for-researchers/open-research-data-clinical-trials-and-public-health-interventions/).

AC11 Removal, Use or Storage of Human Tissue

Award holders whose research involves the removal, use or storage of human tissue as specified in the relevant legislation must:

-comply with the appropriate legislation, ie the Human Tissue Act 2004 and/or the Human Tissue (Scotland) Act 2006;

-follow the relevant standards and Codes of Practice issued by the Human Tissue Authority (HTA) (the MRC Regulatory Support Centre https://mrc.ukri.org/research/facilities-and-resources-for-researchers/regulatory-support-centre/ has summarised these);

-follow the MRC guidance detailed within the Policies and Guidance for Researchers https://mrc.ukri.org/research/policies-and- guidance-for-researchers/, to download the Human Tissue and Biological Samples for Use in medical Research PDF.

Where research involves the use of human tissues and cells to treat patients (human application), award holders must also:

For peer review only

-comply with the Human Tissue (Quality and Safety for Human Application) Regulations 2007;

-work within the applicable regulations and standards as dictated by the Human Tissue Authority, Medicines and Healthcare products Regulatory Agency (MHRA), Human Fertilisation and Embryology Authority and Health Research Authority. The UK Stem Cell Tool Kit (www.sc-toolkit.ac.uk/home.cfm), gives guidance on applicable regulatory routes, and the MHRA Innovation Office (www.gov.uk/government/groups/mhra-innovation-office), provides a regulatory advice service for regenerative medicine.

When research involves the use of human fetal tissue, or non-fetal products of conception (ie amniotic fluids, umbilical cord, placenta or membranes), researchers should follow the guidance set out in Consent Code of Practice issued by the HTA (in particular, please see paragraphs 141-143 at https://www.hta.gov.uk/hta-codes-practice-and-standards-0).

When research involves procedures for the removal of human tissue at post-mortem examination, researchers must also follow guidance issued by the Health Departments and Local Health Authorities.

AC12 Stem Cells

Award holders whose research involves human stem cell lines (both embryonic and adult) must:

-Abide by the UK Code of Practice for the use of Human Stem Cell lines (Code of Practice can be downloaded from the MRC website: https://mrc.ukri.org/research/policies-and-guidance-for-researchers/uk-stem-cell-bank-steering-committee/).
-Ensure that they hold all relevant licenses, accreditations and approvals from, and abide by the Codes of Practice issued by, but not limited to, the Human Fertilisation and Embryology Authority (HFEA; see AC10), the Human Tissue Authority (HTA; see AC12), the Health Research Authority (HRA; for research ethics, gene therapy and confidentiality; see AC6, AC7, AC8), the Medicines and Healthcare products Regulatory Agency (MHRA; see AC6, AC7, AC8), the EU Tissue and Cells Directive (where applicable).

In the case of research involving human embryonic stem cells:

-Deposit a sample of every human embryonic stem cell line derived with MRC funding in the UK Stem Cell Bank; applications to deposit or access banked stem cell lines must be approved by the Steering Committee for the UK Stem Cell Bank and for the Use of Stem Cell Lines (https://mrc.ukri.org/research/policies-and-guidance-for-researchers/uk-stem-cell-bank-steering-committee/).

-Not pass samples of human embryonic stem cell lines to third parties other than those approved by the Steering Committee for the UK Stem Cell Bank and for the Use of Stem Cell Lines and/or the HFEA.

-Not take human embryonic stem cell lines out of the UK unless approved by the Steering Committee for the UK Stem Cell Bank and for the Use of Stem Cell Lines and/or the HFEA.

-Scientists from overseas wishing to conduct human embryonic stem cell research in the UK as visiting workers must provide a written statement from their home institution, outlining that as the employer of the visiting worker they take on the responsibilities of ensuring their employee works to and complies with the requirements of the UK Governance landscape, set out in the UK Code of Practice.

-Send copies of publications to the UK Stem Cell Bank, and agree that the UK Stem Cell Bank may post summaries of published results on their web site.

-Assist the MRC and the UK Stem Cell Bank, on request, with public engagement activities.

AC13 Human Fertilisation

When research involves the use of human gametes, embryos or human admixed embryos researchers must act in accordance with the Human Fertilisation and Embryology Act 1990 as amended in 2008 and 2015 (the Human Fertilisation and Embryology (Mitochondrial Donation) Regulations). This includes obtaining a research licence to undertake activities covered by the Act.
Further information can be obtained from https://www.hfea.gov.uk/.

AC14 Ionising radiation

Under the Ionising Radiation (Medical Exposure) Regulations 2017 and the Ionising Radiation (Medical Exposure) (Northern Ireland) Regulations 2018, Research Ethics Committee approval is required where

For peer review only - http://bmjopen.bmj.com/site/about/guidelines.xhtml

participants are to be exposed to ionising radiation as part of their involvement in medical or biomedical, diagnostic or therapeutic, research.

Research studies involving the administration of radioactive substances must also be approved by the Administration of Radioactive Substances Advisory Committee (ARSAC).

For further guidance (https://www.hra.nhs.uk/planning-and-improving-research/policies-standards-legislation/ionising-radiation/).

AC15 Genetic Modification

In accordance with the Genetically Modified Organisms (Contained Use) Regulations 2014, research organisations and individuals undertaking genetic modification must be registered with the Health and Safety Executive (HSE), undertake risk assessment and seek consent where appropriate.

Researchers who carry out genetic modification should be familiar with the legislative requirements and with the Scientific Advisory Committee on Genetic Modification (Contained Use) guidance. Advice can be obtained from HSE Head Office or from your nearest HSE Office and Knowledge Centre (https://www.hse.gov.uk/contact/maps/index.htm).

AC16 Dangerous Pathogens

Research organisations accommodating projects involving the use of dangerous pathogens must comply with the safeguards recommended by the Advisory Committee on Dangerous Pathogens (https://www.hse.gov. uk/aboutus/meetings/committees/acdp/index.htm) in their guidance 'Infection at work: controlling the risk' (https://www.hse.gov. uk/biosafety/infection.htm), 'Biological Agents: the principles, design and operation of containment in a level 4 facility' (https://www. hse.gov.uk/biosafety/information.htm).

AC17 Controlled Drugs and Substances

When research requires the use of one or more of the drugs controlled under the Psychoactive Substances Act 2016 or the Misuse of Drugs Act, 1971 and its subsequent amendments, researchers must hold an appropriate Home Office licence in accordance with the most up to date Regulations.

AC18 Open Access Policy - Publication Repositories

To comply with the UKRI Policy on Open Access (see RGC 12.4 of the UKRI Research Council fEC Terms and Conditions) the MRC requires all publications to be deposited at the earliest opportunity, and certainly within six months of publication, in Europe PubMed Central (europepmc.org/). This applies both during and after the period of funding. The condition is subject to compliance with publishers' copyright and licensing policies. Whenever possible, the article deposited should be the published version. For more information see (https://mrc.ukri.org/research/policies-and-guidance-for-researchers/open-access-policy/).

AC19 MRC Industry Collaboration Agreement

It is a condition of MRC Industry Collaboration Agreement (MICA) awards that the PI/research organisation must provide MRC Head Office with a copy of the collaboration agreement, signed by all partners, within 3 months of the date of this letter and prior to the award start date. The agreement must be consistent with the Heads of Terms submitted with the application. The grant cannot be activated, and payments, made until this document has been submitted and approved by the MRC.

For peer review only

ANNEX 1

Summary of Financial Expenditure (COVID-19 Rapid Response Award)

Grant Reference Number: MC_PC_19075

Research Organisation: University of Edingburgh

Principal Investigator: Prof Aziz Sheikh

Summary breakdown of expenditure

**Fund Heading**		**£**
UK Costs	Staff	0.00
	Travel and Subsistence	0.00
	Other	0.00
	Indirect/Estate Costs	0.00
	Sub Total	0.00
Overseas Costs	Staff	0.00
	Travel and Subsistence	0.00
	Other	0.00
	Infrastructure	0.00
	Sub Total	0.00
Total		**0.00**
**Award Value**		0.00
Balance to repay		0.00

I confirm all monies have been used for the purpose intended as stated in the awarded application.

Signed Principal Investigator: ……………………….

For peer review only

Early pandemic evaluation and enhanced surveillance of COVID-19 (EAVE II): protocol for an observational study using linked Scottish national data

Journal:	*BMJ Open*
Manuscript ID	bmjopen-2020-039097.R1
Article Type:	Protocol
Date Submitted by the Author:	25-May-2020

Complete List of Authors:	Simpson, Colin; Victoria University of Wellington, Wellington School of Health, Faculty of Health; The University of Edinburgh, Usher Institute<br>Robertson, Chris; University of Strathclyde, Department of Mathematics and Statistics; Public Health Scotland<br>Vasileiou, Eleftheria; University of Edinburgh, Usher Institute ;<br>McMenamin, Jim; Public Health Scotland<br>Gunson, Rory; West Of Scotland Specialist Virology Centre<br>Ritchie, Lewis; University of Aberdeen, Centre of Academic Primary Care<br>Woolhouse, Mark; The University of Edinburgh, Usher Institute<br>Morrice, Lynn; The University of Edinburgh, Usher Institute<br>Kelly , Dave; Albasoft Ltd, The Centre for Health Science<br>Stagg, Helen R.; The University of Edinburgh, Usher Institute<br>Marques, Diogo; Public Health Scotland<br>Murray, Josie; Public Health Scotland<br>Sheikh, Aziz; The University of Edinburgh, Usher Institute

**Primary Subject Heading**:	Infectious diseases
Secondary Subject Heading:	Infectious diseases, Epidemiology, Public health
Keywords:	Public health < INFECTIOUS DISEASES, Epidemiology < INFECTIOUS DISEASES, PUBLIC HEALTH, RESPIRATORY MEDICINE (see Thoracic Medicine)

I, the Submitting Author has the right to grant and does grant on behalf of all authors of the Work (as defined in the below author licence), an exclusive licence and/or a non-exclusive licence for contributions from authors who are: i) UK Crown employees; ii) where BMJ has agreed a CC-BY licence shall apply, and/or iii) in accordance with the terms applicable for US Federal Government officers or employees acting as part of their official duties; on a worldwide, perpetual, irrevocable, royalty-free basis to BMJ Publishing Group Ltd ("BMJ") its licensees and where the relevant Journal is co-owned by BMJ to the co-owners of the Journal, to publish the Work in this journal and any other BMJ products and to exploit all rights, as set out in our licence.

The Submitting Author accepts and understands that any supply made under these terms is made by BMJ to the Submitting Author unless you are acting as an employee on behalf of your employer or a postgraduate student of an affiliated institution which is paying any applicable article publishing charge ("APC") for Open Access articles. Where the Submitting Author wishes to make the Work available on an Open Access basis (and intends to pay the relevant APC), the terms of reuse of such Open Access shall be governed by a Creative Commons licence – details of these licences and which Creative Commons licence will apply to this Work are set out in our licence referred to above.

Other than as permitted in any relevant BMJ Author's Self Archiving Policies, I confirm this Work has not been accepted for publication elsewhere, is not being considered for publication elsewhere and does not duplicate material already published. I confirm all authors consent to publication of this Work and authorise the granting of this licence.

For peer review only

Early pandemic evaluation and enhanced surveillance of COVID-19 (EAVE II): protocol for an observational study using linked Scottish national data

Prof. Colin R. Simpson, Wellington School of Health, Faculty of Health, Victoria University of Wellington, Wellington, New Zealand and Usher Institute, The University of Edinburgh, Edinburgh, UK

Prof. Chris Robertson, Department of Mathematics and Statistics, University of Strathclyde, Glasgow, UK and Public Health Scotland, Glasgow, UK

Dr Eleftheria Vasileiou, Usher Institute, The University of Edinburgh, Teviot, Medical School, Edinburgh, UK, EH8 9AG, eleftheria.vasileiou@ed.ac.uk, +44 07732961139 (corresponding author)

Dr Jim McMenamin, Public Health Scotland, Meridian Court, 5 Cadogan Street, Glasgow, UK

Prof. Rory Gunson, West of Scotland Specialist Virology Centre, Glasgow, UK

Sir Lewis D. Ritchie, Centre of Academic Primary Care, University of Aberdeen, Aberdeen, UK

Prof. Mark Woolhouse, Usher Institute, The University of Edinburgh, Edinburgh, UK

Dr Lynn Morrice, Usher Institute, The University of Edinburgh, Edinburgh, UK

Dave Kelly, Albasoft Ltd, The Centre for Health Science, Raigmore, Inverness, UK

Dr Helen R. Stagg, Usher Institute, The University of Edinburgh, Edinburgh, UK

Diogo Marques, Public Health Scotland, Meridian Court, 5 Cadogan Street, Glasgow, UK

Josie Murray, Public Health Scotland, Meridian Court, 5 Cadogan Street, Glasgow, UK

Prof. Aziz Sheikh, Usher Institute, The University of Edinburgh, Edinburgh, UK

Keywords: COVID-19, coronavirus, pandemic, surveillance, serology

Word count: 3,281

For peer review only

ABSTRACT

Introduction

Following the emergence of the novel coronavirus SARS-CoV-2 in December 2019 and the ensuing COVID-19 pandemic, population-level surveillance and rapid assessment of the effectiveness of existing or new therapeutic or preventive interventions are required to ensure that interventions are targeted to those at highest risk of serious illness or death from COVID-19. We aim to re-purpose and expand an existing pandemic reporting platform to determine the attack rate of SARS-CoV-2, the uptake and effectiveness of any new pandemic vaccine (once available), and any protective effect conferred by existing or new antimicrobial drugs and other therapies.

Methods and analysis

A prospective observational cohort will be used to monitor daily/weekly the progress of the COVID-19 epidemic and to evaluate the effectiveness of therapeutic interventions in approximately 5.4 million individuals registered in general practices across Scotland. A national linked dataset of patient-level primary care data, out-of-hours, hospitalisation, mortality and laboratory data will be assembled. The primary outcomes will measure association between: a) laboratory confirmed SARS-CoV-2 infection, morbidity and mortality and demographic, socioeconomic and clinical population characteristics; and b) healthcare burden of COVID-19 and demographic, socioeconomic and clinical population characteristics. The secondary outcomes will estimate: a) the uptake (for vaccines only); b) effectiveness; and c) safety of new or existing therapies, vaccines and antimicrobials against SARS-CoV-2 infection. The association between population characteristics and primary outcomes will be assessed via multivariate logistic regression models. The effectiveness of therapies, vaccines and antimicrobials will be assessed from time-dependent Cox models or Poisson regression models. Self-controlled study designs will be explored to estimate the risk of therapeutic and prophylactic-related adverse events.

Ethics and dissemination

We obtained approval from the National Research Ethics Service Committee, Southeast Scotland 02. The study findings will be presented at international conferences and published in peer-reviewed journals.

For peer review only

For peer review only - http://bmjopen.bmj.com/site/about/guidelines.xhtml

Strengths and limitations of this study

- We plan to interrogate national data on the Scottish general population.
- We are expanding an existing national pandemic reporting platform, which uses anonymised individual patient-level data from general practices, hospitals, death registry, virology (reverse transcriptase polymerase chain reaction RT-PCR) and serology tests to investigate the epidemiology of COVID-19 and assess the effectiveness of existing or future preventive and treatment measures.
- This is an observational study therefore insufficient adjustment for confounding, either due to insufficiently granular variable measurement or a lack of variable measurement is a potential concern.

For peer review only

For peer review only - http://bmjopen.bmj.com/site/about/guidelines.xhtml

INTRODUCTION

In the last two centuries, six pandemics (global epidemics) have emerged due to novel influenza and coronavirus strains. During the 20th century, influenza caused three pandemics (1918-19, 1957-58, 1968-69), resulting in millions of clinical cases and deaths.[1-4] An estimated 20-50 million deaths were reported during the 1918-19 influenza pandemic. Fewer (between 1-4 million deaths) were estimated for the 1957-58 and 1968-69 influenza pandemics, respectively.[1-4] The high mortality rates observed in the 20th century against the H1N1, H2N2 and H3N2 influenza viruses were mainly due to lack of prophylactic and therapeutic interventions, such as influenza vaccines and anti-viral medications.[1-4] By comparison, the first pandemic of the 21st century arose from a novel coronavirus, severe acute respiratory syndrome (SARS-CoV), which emerged in 2002-03.[5] SARS caused more than 8,000 infections and 700 deaths globally.[2, 5] In 2009-10, the fourth recorded influenza pandemic due the influenza A (H1N1) subtype emerged in Mexico, resulting in more than 200,000 deaths globally and approximately of 11% to 21% the global population infected.[2, 6] Previous exposure to seasonal influenza vaccination induced little or no cross-reactive antibody responses.[7] Particularly low immunological protection against the virus was observed in the younger population (<30 years old) compared to older adults.[7]

In December 2019, a novel coronavirus-SARS coronavirus 2 (SARS-CoV-2)- emerged in Wuhan, China.[8-9] In the space of four months, this virus has now spread globally. The World Health Organization (WHO) declared the coronavirus outbreak a Public Health Emergency of International Concern on 30 January 2020 and then a pandemic on 11 March 2020, as a result of the worldwide spread of the COVID-19 disease.[9] As of 3 April 2020, the WHO has reported more than 970,000 confirmed infections globally and over 50,000 deaths.[9] The elderly, people with underlying medical conditions and people with poor immune function and long-term users of immunosuppressive agents are particularly vulnerable to SARS-CoV-2 and at risk of severe coronavirus-related illness.[8-11] Current data suggest that SARS-CoV-2 has a lower mortality rate, ranged between 0.25% to 3%, than for SARS-CoV (10%) and Middle East Respiratory Syndrome-related coronavirus (MERS-CoV) (37%), respectively.[12-13] It has been postulated (using data from case-studies) that the main driver of disease severity amongst younger patients for COVID-19 are immunopathological lesions, resulting from an excessive pro-inflammatory host response or cytokine storm.[14-15] Amongst older people, an impaired interferon pathway and systemic virus dissemination beyond the respiratory tract may lead to severe disease.[14-15] The absence of immunity from historic exposure to existing seasonal vaccination or anti-viral therapy also (in comparison to influenza) renders COVID-19 a significant global health threat, which demands an urgent response from national and international agencies.

Rapid large observational epidemiological studies are now required to identify the epidemiological and clinical profile of the COVID-19 pandemic. These studies can also be used to estimate the effectiveness of any existing or new healthcare interventions, such as vaccines and anti-viral therapies (e.g. the introduction of any new pandemic vaccine), where it is unethical and/or not feasible to mount more rigorous experimental studies.

For peer review only - http://bmjopen.bmj.com/site/about/guidelines.xhtml

Using linked routine sources of primary, secondary, mortality and virological/serological testing data, this study aims to describe the epidemiology of COVID-19 in Scotland and in due course help establish the effectiveness of existing or new therapeutic interventions against the coronavirus that are not subjected to formal clinical trials. Specifically, our objectives are to:

Primary objectives

a) Determine the epidemiological risk factors for infection, morbidity, mortality of COVID-19 (e.g. laboratory and serology confirmed SARS_CoV-2 infection in relation to demographic, socioeconomic and clinical population characteristics);

b) Determine the healthcare burden of COVID-19 (e.g. COVID-19-related morbidity and mortality in relation to demographic, socioeconomic and clinical population characteristics);

Secondary objectives

a) Measure the uptake of prophylactic interventions (e.g. vaccines);

b) Estimate the effectiveness of any new or existing of prophylactic and therapeutic interventions (e.g. new or repurposed therapies, vaccines, antimicrobials);

c) Assess the safety of any new or existing of prophylactic and therapeutic interventions (e.g. new or repurposed therapies, vaccines, antimicrobials).

This work will re-purpose and expand the hibernated Early Estimation of Vaccine and Anti-Viral Effectiveness (EAVE) project part of the NIHR Pandemic Preparedness Research Portfolio [16][17], and a proven platform for studies on seasonal and pandemic influenza vaccine and anti-viral assessment.[17-21]

METHODS

Study design and population

We will undertake a timely analysis of a large national open prospective observational cohort of patients using a unique community, hospital and laboratory linked dataset. We will seek to extract data on 5.4 million people from across Scotland (Figure 1). Therefore, our study aims to collect data from all residents in Scotland registered with a general practice which translates to over 91% coverage of the Scottish population.[21]

Databases

Individual-level data from general practices will be extracted and linked deterministically to secondary and laboratory healthcare datasets using the Community Health Index (CHI).[17] The CHI number is a unique identifier provided by the National Health Service (NHS) for each resident in Scotland registered with a general practice. A CHI number is also allocated to patients that may have no number when present for treatment as the CHI number is mandatory for all clinical communications. Thus, non-Scottish patients and other temporary residents can also have a CHI number allocated, if required however wherever possible temporary patients will be excluded from this analysis. [22] The linkage of the datasets and analysis will take place within a secure Trusted Research Environment (TRE).[17]

For peer review only - http://bmjopen.bmj.com/site/about/guidelines.xhtml

Primary care

Almost all individuals in Scotland are registered with a general practice, which provide free of charge healthcare services. Data from all patients registered in general practices will be extracted and studied. The University of Edinburgh and Public Health Scotland (PHS) will recruit the additional general practices through Albasoft Ltd.[17-21] Albasoft Ltd is the trusted third party that will carry out the data extraction from all general practices using the Enhanced Services Contract Reporting Options (ESCRO) system.[17-21] We will also extract data from a network of COVID-19 Community Hubs and Assessment Centres established by NHS Health Boards across Scotland.[23] The aim of this network is to provide a direct and rapid route of people with COVID-19 symptoms that have worsened or not improved after a week. Patients can call NHS 24 for an initial assessment and then if needed the call will be passed to a telephone Community Hub, staffed by clinical decision makers.[23] The clinical decision maker will then decide if an appointment for a face to face consultation at an Assessment Centre is necessary.[23] Previous observational studies have shown over 91% completeness of capture of contacts and accuracy of clinical event coding (Read codes) among practices in Scotland.[21]

Secondary care

The Scottish Morbidity Record (SMR) database will be used to derive information for all in-patient hospitalisations and emergency admissions in Scotland, which is maintained by the Information Services Division (ISD).[24] Specifically, we will use data from the SMR01 record which is an episode-based patient record for all inpatients and day cases discharged from non-obstetric and non-psychiatric specialties in Scotland.[25] Data from the SMR02 record will also be used, which is an episode based patient record for all inpatients and day cases from Obstetric specialties in the NHS Scotland.[26] The SMR dataset also contains mortality data which derive from the National Records of Scotland (NRS).[27] Regular validation checks are applied to the SMR database. The latest data quality assessment of these SMR datasets have shown over 90% completeness and accuracy in consistency with previous years.[28] We will also extract and link data on prescribing and administration of medicines for inpatients which are available from Scottish Hospital Electronic Prescribing and Medicines Administration (HEPMA) systems.[29] The study data will also be linked with data from patients admitted to adult general Intensive Care Units (ICU) which derive from the Scottish Intensive Care Society Audit Group (SICSAG) national database.[30] The database contains detailed information on the management of critically ill or injured patients. Data are collected from all general ICU and combined ICU/High Dependency Units (HDU). Data from more than 90% general HDUs and a number of specialist ICUs and HDUs are collected by the database.[30]

Laboratory and serology data

The Electronic Communication of Surveillance in Scotland (ECOSS) system of PHS is a database that holds surveillance data on various microorganisms (e.g. influenza virus, coronavirus) and infections reported from diagnostics and reference laboratories.[31] Data on laboratory results for all reverse transcriptase polymerase chain reaction (RT-PCR) tests carried out in Scotland are being collated by ECOSS and can be linked to other data sources).[31]

For peer review only - http://bmjopen.bmj.com/site/about/guidelines.xhtml

Positive laboratory swab samples for SARS-CoV-2 will also be sent to national sequencing centres where 500 SARS-CoV-2 genome sequences will be performed.

In a sub-study, the West of Scotland Specialist Virology Laboratory will collect and store residual sera from routine blood tests from patients until the serology test becomes available.[32] The EAVE study has already stored 1,000 biochemistry samples from a subset of participating practices from 2014, demonstrating that a potential mechanism for the collection and storage of the residual sera work.[17] We aim to collect and store serially throughout the duration of the COVID-19 pandemic. This will be used to determine exposure to SARS-CoV-2 and other viruses by the presence of antibodies).[17]

Exposure definitions and potential confounding factors

The following exposure variables will be used in relation to study's primary outcomes: sex, age, socioeconomic status (SES) and clinical at-risk group. SES will be determined based on the Scottish Multiple Deprivation Index (SIMD). The SIMD classification is based on deprivation quintiles. Quintile 1 refers to the most deprived and quintile 5 refers to the least deprived. The SIMD is a combination of 38 indicators of the following seven domains: income, employment, health, education, housing, geographical access to services and crime.[21] Clinical at-risk groups refer to individuals with certain underlying medical conditions where are at-risk of COVID-related complications and for whom seasonal influenza vaccination is recommended. The following clinical at-risk conditions will be considered: a) chronic respiratory disease (with chronic obstructive pulmonary disease and asthma as subsets); b) chronic heart disease; c) chronic liver disease; d) chronic kidney disease; e) chronic liver disease; f) chronic neurological disease; g) diabetes type 1 and 2; h) conditions or medications causing impaired immune function; i) pregnancy; j) asplenia or dysfunction of spleen; k) obesity (body mass index (BMI) < 20, 20-25, 25-30, 30-39, ≥ 40 kg/m^2) l) hypertension (subsets controlled/uncontrolled hypertension); m) tuberculosis and n) multimorbidity.[21] This list will be updated as more evidence arises within the medical literature. The following exposure variables will be used in relation to study's secondary outcomes: any new vaccines against SARS-CoV-2 and existing or new therapies and antimicrobial medication against COVID-19. These will be determined once our study data are available and any new therapies, vaccines and antimicrobials specifically against the SARS-CoV-2 virus have been produced.

A number of aforementioned and additional population characteristics below will also be used as potential confounding factors in relation to the study's primary and secondary outcomes. Charlson Comorbidity Index will represent the weighted comorbidity score based on secondary care data.[17-21] The urban/rural location will be determined based on the urban/rural 8 fold classification (UR8). The UR8 is the definition of rural areas in Scotland; 1 is assigned to large urban areas and 8 is assigned to remote rural areas.[21] Smoking status will be determined and presented into the following four categories: Current smoker, non-smoker, ex-smoker and not recorded for patients with no data on smoking.[17-21] The type of smoking products (e.g. vaping products) and alcohol use will also be determined, if possible. Previous healthcare usage will be used to measure number of primary care consultations and secondary care admissions in previous years. The number of prescriptions will also be determined for previous years.[17-21] General practice will also be used to account the effect of clustering within practices. The

For peer review only - http://bmjopen.bmj.com/site/about/guidelines.xhtml

effect of population density will also be investigated. Additional exposures such as number of household members for those with a confirmed SARS-CoV-02 infection, daily protective measures will also be investigated given the high transmission rate of COVID-19.

Outcome definitions

The primary outcomes of this study will include: a) laboratory confirmed SARS-CoV-2; b) serum from blood samples taken from biochemistry tests (or rapid antibody tests if available) will be used to determine exposure to SARS-CoV-2 infection by the presence of antibodies; and c) SARS-CoV-2 infection related clinical outcomes including general practice, COVID centres and out-of-hours consultations, hospital admissions including secondary bacterial infections, emergency admissions, out of hours consultations and deaths. Secondary outcomes include: a) vaccine uptake proportions; b) prevention and reduction of SARS-CoV-2 infection-related general practice consultations, hospital admissions including secondary bacterial infections, emergency admissions, out of hours consultations and deaths due to therapies, vaccines and antimicrobials; and c) adverse events related to therapies – e.g. vaccine, antimicrobial administration or other therapies.

Statistical analysis

Baseline characteristics of all study participants will be described in relation to the study's exposures and outcomes of interest. Mean, median, proportions, odds ratios (ORs) and rate ratios (RRs), together with a measure of dispersion will be provided where appropriate to describe differences between the various study groups based on the nature of each variable. The amount of missing data will be described for each variable. Two-tailed hypotheses tests with a 5% significance level will be used for all study's outcomes. All analyses will be carried out using the R statistical programming language.[17-21]

Primary analyses

Epidemiology and healthcare burden of COVID-19

We will determine the epidemiological risk factors such as demographic, socioeconomic and clinical population characteristics in relation to laboratory and serology confirmed SARS-CoV-2 infection. The healthcare burden of COVID-19 in terms of morbidity and mortality in relation to to demographic, socioeconomic and clinical population characteristics will also be determined. SARS-CoV-2 infection will be confirmed via laboratory (RT-PCR) and serology testing. Healthcare burden will be measured via general practice consultations, out-of-hours consultations, A&E attendances, hospital admissions including secondary bacterial infections and deaths. Exposure of interest as per our objectives a and b will change over time as the medical literature and surveillance reporting is continuously updated. Currently, particularly factors of interest for Scotland include: age; sex, geographical location, socioeconomic status, underlying condition or medication and BMI. Analytical techniques including descriptive analysis, univariable and multivariable logistic regression will be used to determine the association between different exposure variables and the likelihood (odds) of the study's primary outcomes (SARS-CoV-2 infection, morbidity, mortality and healthcare burden). The

For peer review only - http://bmjopen.bmj.com/site/about/guidelines.xhtml

effect of confounders and effect modifiers will be explored through causal frameworks generated for each hypothesis,[33] with clinical input.

Secondary analyses

Vaccine uptake

Differences in vaccine uptake will be measured in relation to demographic, socioeconomic and clinical population characteristics. As per primary analyses, exposure of interest will change over time as the medical literature and surveillance reporting is continuously updated. Key sociodemographic and clinical factors will be analysed including age, sex, socioeconomic status and underlying condition. Analytical techniques including univariable and multivariable logistic regression will be used to determine the association between different exposure variables and vaccine uptake. The effect of confounders and effect modifiers will be explored through causal frameworks generated for each hypothesis,[33] with clinical input. Key confounding factors will include age, sex, socioeconomic status and underlying condition. The number of individuals that refuse to be vaccinated and the reasons for declining vaccination will also be investigated, if possible.

Effectiveness of new or existing prophylactic and therapeutic interventions

We will assess the effectiveness of any new or repurposed therapies, vaccines and antimicrobials against SARS-CoV-2-related morbidity and mortality such as general practice and out of hours consultations, hospitalisations including secondary bacterial infections, emergency admissions and deaths. Exposure of interest (therapies, vaccines and antimicrobials) will change over time as the medical literature and surveillance reporting is continuously updated. The proportion of SARS-CoV-2-related clinical outcomes and deaths will be estimated between vaccinated and unvaccinated cases. Vaccine effectiveness (VE) and 95% CIs will be calculated using the formula, VE = (1-Risk Ratio)*100 for unadjusted and adjusted VE estimates. A time-dependent Cox model or the equivalent Poison regression models (taking into account the time at risk and the possibility of multiple events (not for death)) will provide the RRs and 95% CIs of VE for prevention of SARS-CoV-2 related clinical outcomes and deaths. Causal frameworks will be generated for each hypothesis,[33] with clinical input. Key confounders for the VE models will include age, sex, socioeconomic status and underlying condition, with vaccination group representing a time-dependent covariate. In these VE models, propensity variables related to vaccine receipt and effect modifiers (e.g. vaccinations, consultations and hospitalisation in the previous season, urban/rural status, smoking status, Charlson Score and pregnancy) will be used to control for the healthy vaccine effect.[17] This is in addition to the demographic variables, which will always be used.

Similar statistical methods will be used to assess the protective effects of therapies and antimicrobials. A binary variable of ever/never exposure to therapies/antimicrobials as an explanatory variable will be included in the VE analyses. The therapy/antimicrobial exposure will be a second time-dependent exposure for consultation, hospitalisation and death rates analysis. We will also consider using a measure of the volume of therapy/antimicrobial exposure (e.g. length or dose of prescription) if the data are adequate. Use of

For peer review only - http://bmjopen.bmj.com/site/about/guidelines.xhtml

therapies/antimicrobials will be included as a covariate in any of our models where primarily assess VE. Alternatively, exposure to the vaccine will be included in any of our models where primarily assess the effect of therapies/antimicrobials, if appropriate. For example, the effect of therapies/antimicrobials may be assessed from a period before the vaccine becomes available and, in such instances, no adjustment needs to be made.

Safety of new or existing prophylactic and therapeutic interventions

We will determine any adverse events following the administration of new or repurposed therapies, vaccines and antimicrobials. Specific therapies, vaccines and antimicrobials against SARS-CoV-2 will be determined as the outbreak unfolds and depending on existing medical literature. The risk of adverse events will be estimated using self-controlled study designs. The main assumption in these study designs is that in case of an adverse event related to prophylactic and therapeutic agent exposure then the occurrence of on adverse event in the period after administration is greater than in periods in the same patients that are temporally not related to prophylactic and therapeutic agent administration.[21] The risk interval (the period at risk for an adverse outcome) and the control interval (the period not at risk for an adverse outcome) will be determined separately for each outcome.[21] Causal frameworks will be generated for each hypothesis,[33] with clinical input. The main advantage of the self-controlled designs is the control for all fixed individual-level confounding since any comparisons are carried out for the same individual rather than between exposed and unexposed populations to therapies, vaccines or antimicrobials.[21] Key confounding and effect modifiers will be determined as the outbreak unfolds and depending on existing medical literature.

Sample size

Our prospective cohort will be constructed from patients registered in all general practices across Scotland with a combined list size of 5.4 million people of all ages. Sample size calculations to assess vaccine and antiviral effectiveness against pandemic influenza have been provided in previous work [17]. Similar sample size calculations are likely to be applicable to the current COVID-19 pandemic, however, sample size calculations (one per key analysis) are dependent on how the COVID-19 outbreak unfolds in Scotland. Thus, our power to answer each objective will be dependent on the frequency of the relevant outcome. Power calculations will be carried out subsequent to the first wave of the pandemic.

Ethics and dissemination

National Research Ethics Service Committee, South East Scotland 02. Findings from this study will be presented at international conferences and published in peer-reviewed journals. Meta-data produced in this study will also become available to Health Data Research UK (HDRUK) Gateway through BREATHE – The Health Data Research Hub for Respiratory Health. STROBE and RECORD (via the COVID-19 extension) will be used to guide transparent reporting.

For peer review only - http://bmjopen.bmj.com/site/about/guidelines.xhtml

Contributors

CRS, CR, JM, LDR, RG and AS contributed to the conception of the study. CRS, CR, EV, JM, RG, LDR, MW, LM, DK, HRS, DM, JM and AS contributed to the study design. CRS, CR, EV, JM, RG, LDR, MW, LM, DK, HRS, DM, JM and AS contributed to drafting the protocol. CRS, CR, EV, JM, RG, LDR, MW, LM, DK, HRS, DM, JM and AS authors revised the manuscript for important intellectual content. CRS, CR, EV, JM, RG, LDR, MW, LM, DK, HRS, DM, JM and AS authors gave final approval of the version to be published.

Acknowledgments

The authors thank and acknowledge Kenny Fraser at Triscribe Ltd and Keith Moffat at Public Health Scotland for his support in this study.

Funding

The EAVE project was funded by the National Institute for Health Research Health Technology Assessment Programme (project number 13/34/14). EAVE II is funded by the Medical Research Council [MR/R008345/1] and supported by the Scottish Government. We also acknowledge the support of HDR UK. HRS is supported by the Medical Research Council [MR/R008345/1].

Disclaimer

The views and opinions expressed therein are those of the authors and do not necessarily reflect those of the Health Technology Assessment programme, NIHR, NHS or the Department of Health.

Competing interests

None declared.

Provenance and peer review

Not commissioned; externally peer reviewed.

Patient and Public Involvement

We will pursue the involvement of patients or the public in our research study.

REFERENCES

1. Kilbourne ED. Influenza pandemics of the 20th century. *Emerg Infect Dis* 2006;12(1):9–14.
2. Bloom DE, Cadarette D. Infectious Disease Threats in the Twenty-First Century: Strengthening the Global Response. *Front Immunol* 2019;10:549.
3. Centers for Disease Control and Prevention. Inlfuenza (Flu). Pandemic Influenza. Past Pandemics. Available from: https://www.cdc.gov/flu/pandemic-resources/basics/past-pandemics.html (accessed March 23, 2020).
4. World Health Organization. Influenza. Past Pandemics. Available from: http://www.euro.who.int/en/health-topics/communicable-diseases/influenza/pandemic-influenza/past-pandemics (accessed March 23, 2020).
5. LeDuc JW, Barry MA. SARS, the First Pandemic of the 21st Century. *Emerg Infect Dis* 2004;10(11):e26.
6. Kelly H, Peck HA, Laurie KL, et al. The age-specific cumulative incidence of infection with pandemic influenza H1N1 2009 was similar in various countries prior to vaccination. *PLoS One* 2011;6(8):e21828.
7. Hancock K, Veguilla V, Lu X et al. Cross-reactive antibody responses to the 2009 pandemic H1N1 influenza virus. *N Engl J Med* 2009;361(20):1945-52.

For peer review only - http://bmjopen.bmj.com/site/about/guidelines.xhtml

8. Adhikari SP, Meng S, Wu YJ, et al. Epidemiology, causes, clinical manifestation and diagnosis, prevention and control of coronavirus disease (COVID-19) during the early outbreak period: a scoping review. *Infect Dis Poverty* 2020;9(1):29.
9. World Health Organization. Coronavirus disease (COVID-19) outbreak. Available from: https://www.who.int/emergencies/diseases/novel-coronavirus-2019 (accessed March 26, 2020).
10. Guo YR, Cao QD, Hong ZS et al. The origin, transmission and clinical therapies on coronavirus disease 2019 (COVID-19) outbreak - an update on the status. *Mil Med Res* 2020;7(1):11.
11. Yang X, Yu Y, Xu J et al. Clinical course and outcomes of critically ill patients with SARS-CoV-2 pneumonia in Wuhan, China: a single-centered, retrospective, observational study. *Lancet Respir Med* 2020;pii:S2213-2600(20)30079-5. [Epub ahead of print]
12. Wilson N, Kvalsvig A, Barnard LT, et al. Case-fatality estimates for COVID-19 calculated by using a lag time for fatality. *Emerg Infect Dis* 2020;26(6).
13. Wang C, Horby PW, Hayden FG et al. A novel coronavirus outbreak of global health concern. *Lancet* 2020;395(10223):470-473.
14. Guan WJ, Ni ZY, Hu Y et al. Clinical Characteristics of Coronavirus Disease 2019 in China. *N Engl J Med* 2020; [Epub ahead of print]
15. Lescure FX, Bouadma L, Nguyen D et al. Clinical and virological data of the first cases of COVID-19 in Europe: a case series. Lancet Infect Dis 2020;
16. Simpson CR, Beever D, Challen K et al. The UK's pandemic influenza research portfolio: a model for future research on emerging infections. Lancet Infect Dis 2019;19(8):e295-e300.
17. Simpson CR, Lone N, McMenamin J, et al. Early estimation of pandemic influenza Antiviral and Vaccine Effectiveness (EAVE): use of a unique community and laboratory national data-linked cohort study. *Health Technol Assess* 2015;19(79).
18. Simpson CR, Ritchie LD, Robertson C et al. Vaccine effectiveness in pandemic influenza - primary care reporting (VIPER): an observational study to assess the effectiveness of the pandemic influenza A (H1N1)v vaccine. *Health Technol Asses* 2010;14:313-46.
19. Simpson CR, Ritchie LD, Robertson C et al. Effectiveness of H1N1 vaccine for the prevention of pandemic influenza in Scotland, UK: a retrospective observational cohort study. *Lancet Infect Dis* 2012;12:696-702.
20. Simpson CR, Lone N, Kavanagh K, et al. Seasonal Influenza Vaccine Effectiveness (SIVE): an observational retrospective cohort study – exploitation of a unique community-based national-linked database to determine the effectiveness of the seasonal trivalent influenza vaccine. *Health Services and Delivery Research* 2013;1(10).
21. Simpson CR, Lone NI, Kavanagh K, et al. Evaluating the effectiveness, impact and safety of live attenuated and seasonal inactivated influenza vaccination: protocol for the Seasonal Influenza Vaccination Effectiveness II (SIVE II) study. *BMJ Open* 2017;7:e014200.

For peer review only - http://bmjopen.bmj.com/site/about/guidelines.xhtml

22. National Services Scotland. Information Services Division. CHI Number. Available from: https://www.ndc.scot.nhs.uk/Dictionary-A-Z/Definitions/index.asp?ID=128&Title=CHI%20Number
23. Scottish Government. COVID-19 community hubs. Available from: https://www.gov.scot/publications/coronavirus-covid-19-clinical-guidance-care-at-home-housing-support-and-sheltered-housing/pages/covid-19-community-hubs/ (accessed April 3, 2020).
24. National Services Scotland. Information Services Division. SMR Record Type. Available from: https://www.ndc.scot.nhs.uk/Dictionary-A-Z/Definitions/index.asp?Search=S&ID=458&Title=SMR%20Record%20Type (accessed March 24, 2020).
25. National Services Scotland. Information Services Division. SMR01 – General/Acute Inpatient and Day Case. Available from: https://www.ndc.scot.nhs.uk/Dictionary-A-Z/Definitions/index.asp?Search=S&ID=460&Title=SMR01%20-%20General/Acute%20Inpatient%20and%20Day%20Case (accessed March 24, 2020).
26. National Services Scotland. Information Services Division. SMR02 – Maternity Inpatient and Day Care. Available from: https://www.ndc.scot.nhs.uk/Dictionary-A-Z/Definitions/index.asp?Search=S&ID=461&Title=SMR02%20-%20Maternity%20Inpatient%20and%20Day%20Case (accessed March 24, 2020).
27. National Services Scotland. Information Services Division. Deaths. Available from: https://www.isdscotland.org/Health-Topics/Deaths/ (accessed March 24, 2020).
28. National Services Scotland. Information Services Division. Data Quality Assurance. Available from: https://www.isdscotland.org/Products-and-Services/Data-Quality/Assessments/index.asp?Co=Y (accessed March 24, 2020).
29. NHS Scotland. eHealth. HEPMA. Available from: https://www.ehealth.scot/case-studies/hepma/ (accessed March 24, 2020).
30. Scottish Intensive Care Society Audit Group. Available from: https://www.sicsag.scot.nhs.uk/about/main.html (accessed March 30, 2020).
31. Health Protection Scotland. Data and surveillance. Available from: https://www.hps.scot.nhs.uk/data/ (accessed March 25, 2020).
32. NHS Greater Glasgow and Clyde. West of Scotland Specialist Virology Centre. Available from: https://www.nhsggc.org.uk/about-us/professional-support-sites/laboratory-medicine/laboratory-disciplines/microbiology-and-virology/west-of-scotland-specialist-virology-centre/# (accessed March 25, 2020).
33. Victora CG, Huttly SR, Fuchs SC, Olinto MT. The role of conceptual frameworks in epidemiological analysis: a hierarchical approach. *Int J Epidemiol* 1997; 26(1): 224-7.

Figure 1. Flow diagram for EAVE II project.
ECOSS: Electronic Communication of Surveillance in Scotland; NHS: National Health Service; HEPMA: Hospital Electronic Prescribing and Medicines Administration; RT-PCR: reverse transcriptase polymerase chain reaction; PHS: Public Health Scotland; eDRIS: The electronic Data Research and Innovation Service; ISD: Information Services Scotland.

For peer review only - http://bmjopen.bmj.com/site/about/guidelines.xhtml

For peer review only

For peer review only - http://bmjopen.bmj.com/site/about/guidelines.xhtml

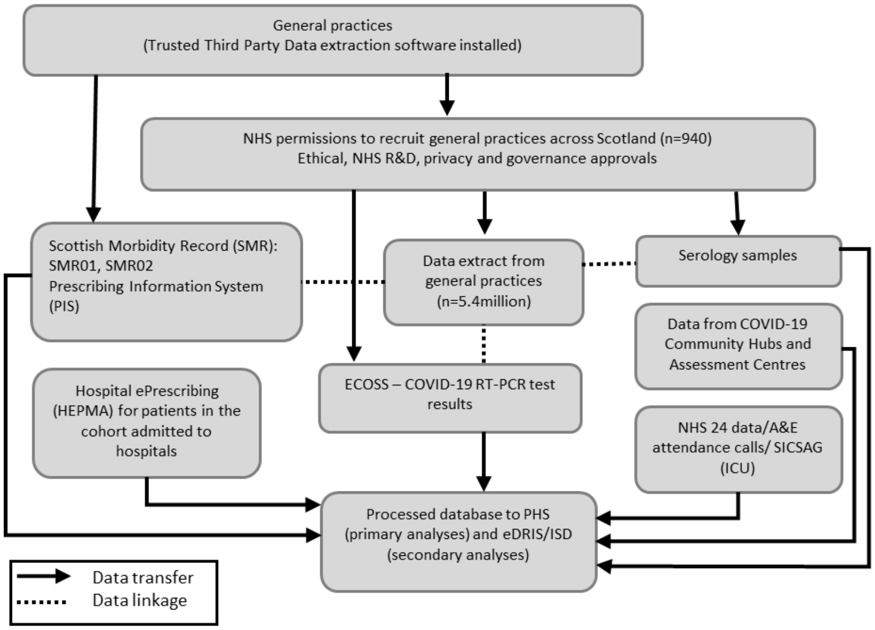

Figure 1. Flow diagram for EAVE II project.
ECOSS: Electronic Communication of Surveillance in Scotland; NHS: National Health Service; HEPMA: Hospital Electronic Prescribing and Medicines Administration; RT-PCR: reverse transcriptase polymerase chain reaction; PHS: Public Health Scotland; eDRIS: The electronic Data Research and Innovation Service; ISD: Information Services Scotland.

90x90mm (300 x 300 DPI)

BMJ Open

Early pandemic evaluation and enhanced surveillance of COVID-19 (EAVE II): protocol for an observational study using linked Scottish national data

Journal:	*BMJ Open*
Manuscript ID	bmjopen-2020-039097.R2
Article Type:	Protocol
Date Submitted by the Author:	01-Jun-2020
Complete List of Authors:	Simpson, Colin; Victoria University of Wellington, Wellington School of Health, Faculty of Health; The University of Edinburgh, Usher Institute<br>Robertson, Chris; University of Strathclyde, Department of Mathematics and Statistics; Public Health Scotland<br>Vasileiou, Eleftheria; University of Edinburgh, Usher Institute ;<br>McMenamin, Jim; Public Health Scotland<br>Gunson, Rory; West Of Scotland Specialist Virology Centre<br>Ritchie, Lewis; University of Aberdeen, Centre of Academic Primary Care<br>Woolhouse, Mark; The University of Edinburgh, Usher Institute<br>Morrice, Lynn; The University of Edinburgh, Usher Institute<br>Kelly , Dave; Albasoft Ltd, The Centre for Health Science<br>Stagg, Helen R.; The University of Edinburgh, Usher Institute<br>Marques, Diogo; Public Health Scotland<br>Murray, Josie; Public Health Scotland<br>Sheikh, Aziz; The University of Edinburgh, Usher Institute
**Primary Subject Heading**:	Infectious diseases
Secondary Subject Heading:	Infectious diseases, Epidemiology, Public health
Keywords:	Public health < INFECTIOUS DISEASES, Epidemiology < INFECTIOUS DISEASES, PUBLIC HEALTH, RESPIRATORY MEDICINE (see Thoracic Medicine)

SCHOLARONE™
Manuscripts

BMJ

I, the Submitting Author has the right to grant and does grant on behalf of all authors of the Work (as defined in the below author licence), an exclusive licence and/or a non-exclusive licence for contributions from authors who are: i) UK Crown employees; ii) where BMJ has agreed a CC-BY licence shall apply, and/or iii) in accordance with the terms applicable for US Federal Government officers or employees acting as part of their official duties; on a worldwide, perpetual, irrevocable, royalty-free basis to BMJ Publishing Group Ltd ("BMJ") its licensees and where the relevant Journal is co-owned by BMJ to the co-owners of the Journal, to publish the Work in this journal and any other BMJ products and to exploit all rights, as set out in our licence.

The Submitting Author accepts and understands that any supply made under these terms is made by BMJ to the Submitting Author unless you are acting as an employee on behalf of your employer or a postgraduate student of an affiliated institution which is paying any applicable article publishing charge ("APC") for Open Access articles. Where the Submitting Author wishes to make the Work available on an Open Access basis (and intends to pay the relevant APC), the terms of reuse of such Open Access shall be governed by a Creative Commons licence – details of these licences and which Creative Commons licence will apply to this Work are set out in our licence referred to above.

Other than as permitted in any relevant BMJ Author's Self Archiving Policies, I confirm this Work has not been accepted for publication elsewhere, is not being considered for publication elsewhere and does not duplicate material already published. I confirm all authors consent to publication of this Work and authorise the granting of this licence.

For peer review only

Early pandemic evaluation and enhanced surveillance of COVID-19 (EAVE II): protocol for an observational study using linked Scottish national data

Prof. Colin R. Simpson, Wellington School of Health, Faculty of Health, Victoria University of Wellington, Wellington, New Zealand and Usher Institute, The University of Edinburgh, Edinburgh, UK

Prof. Chris Robertson, Department of Mathematics and Statistics, University of Strathclyde, Glasgow, UK and Public Health Scotland, Glasgow, UK

Dr Eleftheria Vasileiou, Usher Institute, The University of Edinburgh, Teviot, Medical School, Edinburgh, UK, EH8 9AG, eleftheria.vasileiou@ed.ac.uk, +44 07732961139 (corresponding author)

Dr Jim McMenamin, Public Health Scotland, Meridian Court, 5 Cadogan Street, Glasgow, UK

Prof. Rory Gunson, West of Scotland Specialist Virology Centre, Glasgow, UK

Sir Lewis D. Ritchie, Centre of Academic Primary Care, University of Aberdeen, Aberdeen, UK

Prof. Mark Woolhouse, Usher Institute, The University of Edinburgh, Edinburgh, UK

Dr Lynn Morrice, Usher Institute, The University of Edinburgh, Edinburgh, UK

Dave Kelly, Albasoft Ltd, The Centre for Health Science, Raigmore, Inverness, UK

Dr Helen R. Stagg, Usher Institute, The University of Edinburgh, Edinburgh, UK

Diogo Marques, Public Health Scotland, Meridian Court, 5 Cadogan Street, Glasgow, UK

Josie Murray, Public Health Scotland, Meridian Court, 5 Cadogan Street, Glasgow, UK

Prof. Aziz Sheikh, Usher Institute, The University of Edinburgh, Edinburgh, UK

Keywords: COVID-19, coronavirus, pandemic, surveillance, serology

Word count: 3,319

For peer review only - http://bmjopen.bmj.com/site/about/guidelines.xhtml

ABSTRACT

Introduction

Following the emergence of the novel coronavirus SARS-CoV-2 in December 2019 and the ensuing COVID-19 pandemic, population-level surveillance and rapid assessment of the effectiveness of existing or new therapeutic or preventive interventions are required to ensure that interventions are targeted to those at highest risk of serious illness or death from COVID-19. We aim to re-purpose and expand an existing pandemic reporting platform to determine the attack rate of SARS-CoV-2, the uptake and effectiveness of any new pandemic vaccine (once available), and any protective effect conferred by existing or new antimicrobial drugs and other therapies.

Methods and analysis

A prospective observational cohort will be used to monitor daily/weekly the progress of the COVID-19 epidemic and to evaluate the effectiveness of therapeutic interventions in approximately 5.4 million individuals registered in general practices across Scotland. A national linked dataset of patient-level primary care data, out-of-hours, hospitalisation, mortality and laboratory data will be assembled. The primary outcomes will measure association between: a) laboratory confirmed SARS-CoV-2 infection, morbidity and mortality and demographic, socioeconomic and clinical population characteristics; and b) healthcare burden of COVID-19 and demographic, socioeconomic and clinical population characteristics. The secondary outcomes will estimate: a) the uptake (for vaccines only); b) effectiveness; and c) safety of new or existing therapies, vaccines and antimicrobials against SARS-CoV-2 infection. The association between population characteristics and primary outcomes will be assessed via multivariate logistic regression models. The effectiveness of therapies, vaccines and antimicrobials will be assessed from time-dependent Cox models or Poisson regression models. Self-controlled study designs will be explored to estimate the risk of therapeutic and prophylactic-related adverse events.

Ethics and dissemination

We obtained approval from the National Research Ethics Service Committee, Southeast Scotland 02. The study findings will be presented at international conferences and published in peer-reviewed journals.

For peer review only

For peer review only - http://bmjopen.bmj.com/site/about/guidelines.xhtml

Strengths and limitations of this study

- We plan to interrogate national data on the Scottish general population.
- We are expanding an existing national pandemic reporting platform, which uses anonymised individual patient-level data from general practices, hospitals, death registry, virology (reverse transcriptase polymerase chain reaction RT-PCR) and serology tests to investigate the epidemiology of COVID-19 and assess the effectiveness of existing or future preventive and treatment measures.
- This is an observational study therefore insufficient adjustment for confounding, either due to insufficiently granular variable measurement or a lack of variable measurement is a potential concern.

For peer review only

For peer review only - http://bmjopen.bmj.com/site/about/guidelines.xhtml

INTRODUCTION

In the last two centuries, six pandemics (global epidemics) have emerged due to novel influenza and coronavirus strains. During the 20th century, influenza caused three pandemics (1918-19, 1957-58, 1968-69), resulting in millions of clinical cases and deaths.[1-4] An estimated 20-50 million deaths were reported during the 1918-19 influenza pandemic. Fewer (between 1-4 million deaths) were estimated for the 1957-58 and 1968-69 influenza pandemics, respectively.[1-4] The high mortality rates observed in the 20th century against the H1N1, H2N2 and H3N2 influenza viruses were mainly due to lack of prophylactic and therapeutic interventions, such as influenza vaccines and anti-viral medications.[1-4] By comparison, the first pandemic of the 21st century arose from a novel coronavirus, severe acute respiratory syndrome (SARS-CoV), which emerged in 2002-03.[5] SARS caused more than 8,000 infections and 700 deaths globally.[2, 5] In 2009-10, the fourth recorded influenza pandemic due the influenza A (H1N1) subtype emerged in Mexico, resulting in more than 200,000 deaths globally and approximately of 11% to 21% the global population infected.[2, 6] Previous exposure to seasonal influenza vaccination induced little or no cross-reactive antibody responses.[7] Particularly low immunological protection against the virus was observed in the younger population (<30 years old) compared to older adults.[7]

In December 2019, a novel coronavirus-SARS coronavirus 2 (SARS-CoV-2)- emerged in Wuhan, China.[8-9] In the space of four months, this virus has now spread globally. The World Health Organization (WHO) declared the coronavirus outbreak a Public Health Emergency of International Concern on 30 January 2020 and then a pandemic on 11 March 2020, as a result of the worldwide spread of the COVID-19 disease.[9] As of 3 April 2020, the WHO has reported more than 970,000 confirmed infections globally and over 50,000 deaths.[9] The elderly, people with underlying medical conditions and people with poor immune function and long-term users of immunosuppressive agents are particularly vulnerable to SARS-CoV-2 and at risk of severe coronavirus-related illness.[8-11] Current data suggest that SARS-CoV-2 has a lower mortality rate, ranged between 0.25% to 3%, than for SARS-CoV (10%) and Middle East Respiratory Syndrome-related coronavirus (MERS-CoV) (37%), respectively.[12-13] It has been postulated (using data from case-studies) that the main driver of disease severity amongst younger patients for COVID-19 are immunopathological lesions, resulting from an excessive pro-inflammatory host response or cytokine storm.[14-15] Amongst older people, an impaired interferon pathway and systemic virus dissemination beyond the respiratory tract may lead to severe disease.[14-15] The absence of immunity from historic exposure to existing seasonal vaccination or anti-viral therapy also (in comparison to influenza) renders COVID-19 a significant global health threat, which demands an urgent response from national and international agencies.

Rapid large observational epidemiological studies are now required to identify the epidemiological and clinical profile of the COVID-19 pandemic. These studies can also be used to estimate the effectiveness of any existing or new healthcare interventions, such as vaccines and anti-viral therapies (e.g. the introduction of any new pandemic vaccine), where it is unethical and/or not feasible to mount more rigorous experimental studies.

For peer review only - http://bmjopen.bmj.com/site/about/guidelines.xhtml

Using linked routine sources of primary, secondary, mortality and virological/serological testing data, this study aims to describe the epidemiology of COVID-19 in Scotland and in due course help establish the effectiveness of existing or new therapeutic interventions against the coronavirus that are not subjected to formal clinical trials. Specifically, our objectives are to:

Primary objectives

a) Determine the epidemiological risk factors for infection, morbidity, mortality of COVID-19 (e.g. laboratory and serology confirmed SARS_CoV-2 infection in relation to demographic, socioeconomic and clinical population characteristics);

b) Determine the healthcare burden of COVID-19 (e.g. COVID-19-related morbidity and mortality in relation to demographic, socioeconomic and clinical population characteristics);

Secondary objectives

a) Measure the uptake of prophylactic interventions (e.g. vaccines);

b) Estimate the effectiveness of any new or existing of prophylactic and therapeutic interventions (e.g. new or repurposed therapies, vaccines, antimicrobials);

c) Assess the safety of any new or existing of prophylactic and therapeutic interventions (e.g. new or repurposed therapies, vaccines, antimicrobials).

This work will re-purpose and expand the hibernated Early Estimation of Vaccine and Anti-Viral Effectiveness (EAVE) project part of the NIHR Pandemic Preparedness Research Portfolio [16][17], and a proven platform for studies on seasonal and pandemic influenza vaccine and anti-viral assessment.[17-21]

METHODS

Study design and population

We will undertake a timely analysis of a large national open prospective observational cohort of patients using a unique community, hospital and laboratory linked dataset. We will seek to extract data on 5.4 million people from across Scotland (Figure 1). Therefore, our study aims to collect data from all residents in Scotland registered with a general practice which translates to over 91% coverage of the Scottish population.[21]

Databases

Individual-level data from general practices will be extracted and linked deterministically to secondary and laboratory healthcare datasets using the Community Health Index (CHI).[17] The CHI number is a unique identifier provided by the National Health Service (NHS) for each resident in Scotland registered with a general practice. A CHI number is also allocated to patients that may have no number when present for treatment as the CHI number is mandatory for all clinical communications. Thus, non-Scottish patients and other temporary residents can also have a CHI number allocated, if required however wherever possible temporary patients will be excluded from this analysis. [22] The linkage of the datasets and analysis will take place within a secure Trusted Research Environment (TRE).[17]

For peer review only - http://bmjopen.bmj.com/site/about/guidelines.xhtml

Primary care

Almost all individuals in Scotland are registered with a general practice, which provide free of charge healthcare services. Data from all patients registered in general practices will be extracted and studied. The University of Edinburgh and Public Health Scotland (PHS) will recruit the additional general practices through Albasoft Ltd.[17-21] Albasoft Ltd is the trusted third party that will carry out the data extraction from all general practices using the Enhanced Services Contract Reporting Options (ESCRO) system.[17-21] We will also extract data from a network of COVID-19 Community Hubs and Assessment Centres established by NHS Health Boards across Scotland.[23] The aim of this network is to provide a direct and rapid route of people with COVID-19 symptoms that have worsened or not improved after a week. Patients can call NHS 24 for an initial assessment and then if needed the call will be passed to a telephone Community Hub, staffed by clinical decision makers.[23] The clinical decision maker will then decide if an appointment for a face to face consultation at an Assessment Centre is necessary.[23] Previous observational studies have shown over 91% completeness of capture of contacts and accuracy of clinical event coding (Read codes) among practices in Scotland.[21]

Secondary care

The Scottish Morbidity Record (SMR) database will be used to derive information for all in-patient hospitalisations and emergency admissions in Scotland, which is maintained by the Information Services Division (ISD).[24] Specifically, we will use data from the SMR01 record which is an episode-based patient record for all inpatients and day cases discharged from non-obstetric and non-psychiatric specialties in Scotland.[25] Data from the SMR02 record will also be used, which is an episode based patient record for all inpatients and day cases from Obstetric specialties in the NHS Scotland.[26] The SMR dataset also contains mortality data which derive from the National Records of Scotland (NRS).[27] Regular validation checks are applied to the SMR database. The latest data quality assessment of these SMR datasets have shown over 90% completeness and accuracy in consistency with previous years.[28] We will also extract and link data on prescribing and administration of medicines for inpatients which are available from Scottish Hospital Electronic Prescribing and Medicines Administration (HEPMA) systems.[29] The study data will also be linked with data from patients admitted to adult general Intensive Care Units (ICU) which derive from the Scottish Intensive Care Society Audit Group (SICSAG) national database.[30] The database contains detailed information on the management of critically ill or injured patients. Data are collected from all general ICU and combined ICU/High Dependency Units (HDU). Data from more than 90% general HDUs and a number of specialist ICUs and HDUs are collected by the database.[30]

Laboratory and serology data

The Electronic Communication of Surveillance in Scotland (ECOSS) system of PHS is a database that holds surveillance data on various microorganisms (e.g. influenza virus, coronavirus) and infections reported from diagnostics and reference laboratories.[31] Data on laboratory results for all reverse transcriptase polymerase chain reaction (RT-PCR) tests carried out in Scotland are being collated by ECOSS and can be linked to other data sources).[31]

For peer review only - http://bmjopen.bmj.com/site/about/guidelines.xhtml

Positive laboratory swab samples for SARS-CoV-2 will also be sent to national sequencing centres where 500 SARS-CoV-2 genome sequences will be performed.

In a sub-study, the West of Scotland Specialist Virology Laboratory will collect and store residual sera from routine blood tests from patients until the serology test becomes available.[32] The EAVE study has already stored 1,000 biochemistry samples from a subset of participating practices from 2014, demonstrating that a potential mechanism for the collection and storage of the residual sera work.[17] We aim to collect and store serially throughout the duration of the COVID-19 pandemic. This will be used to determine exposure to SARS-CoV-2 and other viruses by the presence of antibodies).[17]

Exposure definitions and potential confounding factors

The following exposure variables will be used in relation to study's primary outcomes: sex, age, socioeconomic status (SES) and clinical at-risk group. SES will be determined based on the Scottish Multiple Deprivation Index (SIMD). The SIMD classification is based on deprivation quintiles. Quintile 1 refers to the most deprived and quintile 5 refers to the least deprived. The SIMD is a combination of 38 indicators of the following seven domains: income, employment, health, education, housing, geographical access to services and crime.[21] Clinical at-risk groups refer to individuals with certain underlying medical conditions where are at-risk of COVID-related complications and for whom seasonal influenza vaccination is recommended. The following clinical at-risk conditions will be considered: a) chronic respiratory disease (with chronic obstructive pulmonary disease and asthma as subsets); b) chronic heart disease; c) chronic liver disease; d) chronic kidney disease; e) chronic liver disease; f) chronic neurological disease; g) diabetes type 1 and 2; h) conditions or medications causing impaired immune function; i) pregnancy; j) asplenia or dysfunction of spleen; k) obesity (body mass index (BMI) < 20, 20-25, 25-30, 30-39, ≥ 40 kg/m^2) l) hypertension (subsets controlled/uncontrolled hypertension); m) tuberculosis and n) multimorbidity.[21] This list will be updated as more evidence arises within the medical literature. The following exposure variables will be used in relation to study's secondary outcomes: any new vaccines against SARS-CoV-2 and existing or new therapies and antimicrobial medication against COVID-19. These will be determined once our study data are available and any new therapies, vaccines and antimicrobials specifically against the SARS-CoV-2 virus have been produced.

A number of aforementioned and additional population characteristics below will also be used as potential confounding factors in relation to the study's primary and secondary outcomes. Charlson Comorbidity Index will represent the weighted comorbidity score based on secondary care data.[17-21] The urban/rural location will be determined based on the urban/rural 8 fold classification (UR8). The UR8 is the definition of rural areas in Scotland; 1 is assigned to large urban areas and 8 is assigned to remote rural areas.[21] Smoking status will be determined and presented into the following four categories: Current smoker, non-smoker, ex-smoker and not recorded for patients with no data on smoking.[17-21] The type of smoking products (e.g. vaping products) and alcohol use will also be determined, if possible. Previous healthcare usage will be used to measure number of primary care consultations and secondary care admissions in previous years. The number of prescriptions will also be determined for previous years.[17-21] General practice will also be used to account the effect of clustering within practices. The

For peer review only - http://bmjopen.bmj.com/site/about/guidelines.xhtml

effect of population density will also be investigated. Additional exposures such as number of household members for those with a confirmed SARS-CoV-02 infection, daily protective measures will also be investigated given the high transmission rate of COVID-19.

Outcome definitions

The primary outcomes of this study will include: a) laboratory confirmed SARS-CoV-2; b) serum from blood samples taken from biochemistry tests (or rapid antibody tests if available) will be used to determine exposure to SARS-CoV-2 infection by the presence of antibodies; and c) SARS-CoV-2 infection related clinical outcomes including general practice, COVID centres and out-of-hours consultations, hospital admissions including secondary bacterial infections and Multidrug Resistant (MDR) bacteria associated with these infections, emergency admissions, out of hours consultations and deaths. Secondary outcomes include: a) vaccine uptake proportions; b) prevention and reduction of SARS-CoV-2 infection-related general practice consultations, hospital admissions including secondary bacterial infections, emergency admissions, out of hours consultations and deaths due to therapies, vaccines and antimicrobials; and c) adverse events related to therapies – e.g. vaccine, antimicrobial administration or other therapies.

Statistical analysis

Baseline characteristics of all study participants will be described in relation to the study's exposures and outcomes of interest. Mean, median, proportions, odds ratios (ORs) and rate ratios (RRs), together with a measure of dispersion will be provided where appropriate to describe differences between the various study groups based on the nature of each variable. The amount of missing data will be described for each variable. Two-tailed hypotheses tests with a 5% significance level will be used for all study's outcomes. All analyses will be carried out using the R statistical programming language.[17-21]

Primary analyses

Epidemiology and healthcare burden of COVID-19

We will determine the epidemiological risk factors such as demographic, socioeconomic and clinical population characteristics in relation to laboratory and serology confirmed SARS-CoV-2 infection. The healthcare burden of COVID-19 in terms of morbidity and mortality in relation to to demographic, socioeconomic and clinical population characteristics will also be determined. SARS-CoV-2 infection will be confirmed via laboratory (RT-PCR) and serology testing. Healthcare burden will be measured via general practice consultations, out-of-hours consultations, A&E attendances, hospital admissions including secondary bacterial infections and deaths. Exposure of interest as per our objectives a and b will change over time as the medical literature and surveillance reporting is continuously updated. Currently, particularly factors of interest for Scotland include: age; sex, geographical location, socioeconomic status, underlying condition or medication and BMI. Analytical techniques including descriptive analysis, univariable and multivariable logistic regression will be used to determine the association between different exposure variables and the likelihood (odds) of the study's

For peer review only - http://bmjopen.bmj.com/site/about/guidelines.xhtml

primary outcomes (SARS-CoV-2 infection, morbidity, mortality and healthcare burden). The effect of confounders and effect modifiers will be explored through causal frameworks generated for each hypothesis,[33] with clinical input.

Secondary analyses

Vaccine uptake

Differences in vaccine uptake will be measured in relation to demographic, socioeconomic and clinical population characteristics. As per primary analyses, exposure of interest will change over time as the medical literature and surveillance reporting is continuously updated. Key sociodemographic and clinical factors will be analysed including age, sex, socioeconomic status and underlying condition. Analytical techniques including univariable and multivariable logistic regression will be used to determine the association between different exposure variables and vaccine uptake. The effect of confounders and effect modifiers will be explored through causal frameworks generated for each hypothesis,[33] with clinical input. Key confounding factors will include age, sex, socioeconomic status and underlying condition. The number of individuals that refuse to be vaccinated and the reasons for declining vaccination will also be investigated, if possible.

Effectiveness of new or existing prophylactic and therapeutic interventions

We will assess the effectiveness of any new or repurposed therapies, vaccines and antimicrobials against SARS-CoV-2-related morbidity and mortality such as general practice and out of hours consultations, hospitalisations including secondary bacterial infections, emergency admissions and deaths. Exposure of interest (therapies, vaccines and antimicrobials) will change over time as the medical literature and surveillance reporting is continuously updated. The proportion of SARS-CoV-2-related clinical outcomes and deaths will be estimated between vaccinated and unvaccinated cases. Vaccine effectiveness (VE) and 95% CIs will be calculated using the formula, VE = (1-Risk Ratio)*100 for unadjusted and adjusted VE estimates. A time-dependent Cox model or the equivalent Poison regression models (taking into account the time at risk and the possibility of multiple events (not for death)) will provide the RRs and 95% CIs of VE for prevention of SARS-CoV-2 related clinical outcomes and deaths. Causal frameworks will be generated for each hypothesis,[33] with clinical input. Key confounders for the VE models will include age, sex, socioeconomic status and underlying condition, with vaccination group representing a time-dependent covariate. In these VE models, propensity variables related to vaccine receipt and effect modifiers (e.g. vaccinations, consultations and hospitalisation in the previous season, urban/rural status, smoking status, Charlson Score and pregnancy) will be used to control for the healthy vaccine effect.[17] This is in addition to the demographic variables, which will always be used.

Similar statistical methods will be used to assess the protective effects of therapies and antimicrobials. A binary variable of ever/never exposure to therapies/antimicrobials as an explanatory variable will be included in the VE analyses. The therapy/antimicrobial exposure will be a second time-dependent exposure for consultation, hospitalisation and death rates analysis. We will also consider using a measure of the volume of therapy/antimicrobial

For peer review only - http://bmjopen.bmj.com/site/about/guidelines.xhtml

exposure (e.g. length or dose of prescription) if the data are adequate. Use of therapies/antimicrobials will be included as a covariate in any of our models where primarily assess VE. Alternatively, exposure to the vaccine will be included in any of our models where primarily assess the effect of therapies/antimicrobials, if appropriate. For example, the effect of therapies/antimicrobials may be assessed from a period before the vaccine becomes available and, in such instances, no adjustment needs to be made.

Safety of new or existing prophylactic and therapeutic interventions

We will determine any adverse events following the administration of new or repurposed therapies, vaccines and antimicrobials. Specific therapies, vaccines and antimicrobials against SARS-CoV-2 will be determined as the outbreak unfolds and depending on existing medical literature. The risk of adverse events will be estimated using self-controlled study designs. The main assumption in these study designs is that in case of an adverse event related to prophylactic and therapeutic agent exposure then the occurrence of on adverse event in the period after administration is greater than in periods in the same patients that are temporally not related to prophylactic and therapeutic agent administration.[21] The risk interval (the period at risk for an adverse outcome) and the control interval (the period not at risk for an adverse outcome) will be determined separately for each outcome.[21] Causal frameworks will be generated for each hypothesis,[33] with clinical input. The main advantage of the self-controlled designs is the control for all fixed individual-level confounding since any comparisons are carried out for the same individual rather than between exposed and unexposed populations to therapies, vaccines or antimicrobials.[21] Key confounding and effect modifiers will be determined as the outbreak unfolds and depending on existing medical literature.

Sample size

Our prospective cohort will be constructed from patients registered in all general practices across Scotland with a combined list size of 5.4 million people of all ages. Sample size calculations to assess vaccine and antiviral effectiveness against pandemic influenza have been provided in previous work [17]. Similar sample size calculations are likely to be applicable to the current COVID-19 pandemic, however, sample size calculations (one per key analysis) are dependent on how the COVID-19 outbreak unfolds in Scotland. Thus, our power to answer each objective will be dependent on the frequency of the relevant outcome. Power calculations will be carried out subsequent to the first wave of the pandemic.

Patient and public involvement

We will convene a virtual panel of PPI members who will contribute to the interpretation and dissemination of findings.

Ethics and dissemination

This study was approved by the National Research Ethics Service Committee, South East Scotland 02. Findings from this study will be presented at international conferences and published in peer-reviewed journals. Meta-data produced in this study will also become available to Health Data Research UK (HDRUK) Gateway through BREATHE – The Health

For peer review only - http://bmjopen.bmj.com/site/about/guidelines.xhtml

Data Research Hub for Respiratory Health. STROBE and RECORD (via the COVID-19 extension) will be used to guide transparent reporting.

Contributors

CRS, CR, JM, LDR, RG and AS contributed to the conception of the study. CRS, CR, EV, JM, RG, LDR, MW, LM, DK, HRS, DM, JM and AS contributed to the study design. CRS, CR, EV, JM, RG, LDR, MW, LM, DK, HRS, DM, JM and AS contributed to drafting the protocol. CRS, CR, EV, JM, RG, LDR, MW, LM, DK, HRS, DM, JM and AS authors revised the manuscript for important intellectual content. CRS, CR, EV, JM, RG, LDR, MW, LM, DK, HRS, DM, JM and AS authors gave final approval of the version to be published.

Acknowledgments

The authors thank and acknowledge Kenny Fraser at Triscribe Ltd and Keith Moffat at Public Health Scotland for their support in this study.

Funding

The EAVE project was funded by the National Institute for Health Research Health Technology Assessment Programme (project number 13/34/14). EAVE II is funded by the Medical Research Council [MR/R008345/1] and supported by the Scottish Government. We also acknowledge the support of HDR UK. HRS is supported by the Medical Research Council [MR/R008345/1].

Disclaimer

The views and opinions expressed therein are those of the authors and do not necessarily reflect those of the Health Technology Assessment programme, NIHR, NHS or the Department of Health.

Competing interests

None declared.

Provenance and peer review

Not commissioned; externally peer reviewed.

Patient and Public Involvement

We will pursue the involvement of patients or the public in our research study.

REFERENCES

1. Kilbourne ED. Influenza pandemics of the 20th century. *Emerg Infect Dis* 2006;12(1):9–14.
2. Bloom DE, Cadarette D. Infectious Disease Threats in the Twenty-First Century: Strengthening the Global Response. *Front Immunol* 2019;10:549.
3. Centers for Disease Control and Prevention. Inlfuenza (Flu). Pandemic Influenza. Past Pandemics. Available from: https://www.cdc.gov/flu/pandemic-resources/basics/past-pandemics.html (accessed March 23, 2020).
4. World Health Organization. Influenza. Past Pandemics. Available from: http://www.euro.who.int/en/health-topics/communicable-diseases/influenza/pandemic-influenza/past-pandemics (accessed March 23, 2020).
5. LeDuc JW, Barry MA. SARS, the First Pandemic of the 21st Century. *Emerg Infect Dis* 2004;10(11):e26.

For peer review only - http://bmjopen.bmj.com/site/about/guidelines.xhtml

For peer review only

6. Kelly H, Peck HA, Laurie KL, et al. The age-specific cumulative incidence of infection with pandemic influenza H1N1 2009 was similar in various countries prior to vaccination. *PLoS One* 2011;6(8):e21828.
7. Hancock K, Veguilla V, Lu X et al. Cross-reactive antibody responses to the 2009 pandemic H1N1 influenza virus. *N Engl J Med* 2009;361(20):1945-52.
8. Adhikari SP, Meng S, Wu YJ, et al. Epidemiology, causes, clinical manifestation and diagnosis, prevention and control of coronavirus disease (COVID-19) during the early outbreak period: a scoping review. *Infect Dis Poverty* 2020;9(1):29.
9. World Health Organization. Coronavirus disease (COVID-19) outbreak. Available from: https://www.who.int/emergencies/diseases/novel-coronavirus-2019 (accessed March 26, 2020).
10. Guo YR, Cao QD, Hong ZS et al. The origin, transmission and clinical therapies on coronavirus disease 2019 (COVID-19) outbreak - an update on the status. *Mil Med Res* 2020;7(1):11.
11. Yang X, Yu Y, Xu J et al. Clinical course and outcomes of critically ill patients with SARS-CoV-2 pneumonia in Wuhan, China: a single-centered, retrospective, observational study. *Lancet Respir Med* 2020;pii:S2213-2600(20)30079-5. [Epub ahead of print]
12. Wilson N, Kvalsvig A, Barnard LT, et al. Case-fatality estimates for COVID-19 calculated by using a lag time for fatality. *Emerg Infect Dis* 2020;26(6).
13. Wang C, Horby PW, Hayden FG et al. A novel coronavirus outbreak of global health concern. *Lancet* 2020;395(10223):470-473.
14. Guan WJ, Ni ZY, Hu Y et al. Clinical Characteristics of Coronavirus Disease 2019 in China. *N Engl J Med* 2020; [Epub ahead of print]
15. Lescure FX, Bouadma L, Nguyen D et al. Clinical and virological data of the first cases of COVID-19 in Europe: a case series. Lancet Infect Dis 2020;
16. Simpson CR, Beever D, Challen K et al. The UK's pandemic influenza research portfolio: a model for future research on emerging infections. Lancet Infect Dis 2019;19(8):e295-e300.
17. Simpson CR, Lone N, McMenamin J, et al. Early estimation of pandemic influenza Antiviral and Vaccine Effectiveness (EAVE): use of a unique community and laboratory national data-linked cohort study. *Health Technol Assess* 2015;19(79).
18. Simpson CR, Ritchie LD, Robertson C et al. Vaccine effectiveness in pandemic influenza - primary care reporting (VIPER): an observational study to assess the effectiveness of the pandemic influenza A (H1N1)v vaccine. *Health Technol Asses* 2010;14:313-46.
19. Simpson CR, Ritchie LD, Robertson C et al. Effectiveness of H1N1 vaccine for the prevention of pandemic influenza in Scotland, UK: a retrospective observational cohort study. *Lancet Infect Dis* 2012;12:696-702.
20. Simpson CR, Lone N, Kavanagh K, et al. Seasonal Influenza Vaccine Effectiveness (SIVE): an observational retrospective cohort study – exploitation of a unique community-based national-linked database to determine the effectiveness of the seasonal trivalent influenza vaccine. *Health Services and Delivery Research* 2013;1(10).

For peer review only - http://bmjopen.bmj.com/site/about/guidelines.xhtml

21. Simpson CR, Lone NI, Kavanagh K, et al. Evaluating the effectiveness, impact and safety of live attenuated and seasonal inactivated influenza vaccination: protocol for the Seasonal Influenza Vaccination Effectiveness II (SIVE II) study. *BMJ Open* 2017;7:e014200.
22. National Services Scotland. Information Services Division. CHI Number. Available from: https://www.ndc.scot.nhs.uk/Dictionary-A-Z/Definitions/index.asp?ID=128&Title=CHI%20Number
23. Scottish Government. COVID-19 community hubs. Available from: https://www.gov.scot/publications/coronavirus-covid-19-clinical-guidance-care-at-home-housing-support-and-sheltered-housing/pages/covid-19-community-hubs/ (accessed April 3, 2020).
24. National Services Scotland. Information Services Division. SMR Record Type. Available from: https://www.ndc.scot.nhs.uk/Dictionary-A-Z/Definitions/index.asp?Search=S&ID=458&Title=SMR%20Record%20Type (accessed March 24, 2020).
25. National Services Scotland. Information Services Division. SMR01 – General/Acute Inpatient and Day Case. Available from: https://www.ndc.scot.nhs.uk/Dictionary-A-Z/Definitions/index.asp?Search=S&ID=460&Title=SMR01%20-%20General/Acute%20Inpatient%20and%20Day%20Case (accessed March 24, 2020).
26. National Services Scotland. Information Services Division. SMR02 – Maternity Inpatient and Day Care. Available from: https://www.ndc.scot.nhs.uk/Dictionary-A-Z/Definitions/index.asp?Search=S&ID=461&Title=SMR02%20-%20Maternity%20Inpatient%20and%20Day%20Case (accessed March 24, 2020).
27. National Services Scotland. Information Services Division. Deaths. Available from: https://www.isdscotland.org/Health-Topics/Deaths/ (accessed March 24, 2020).
28. National Services Scotland. Information Services Division. Data Quality Assurance. Available from: https://www.isdscotland.org/Products-and-Services/Data-Quality/Assessments/index.asp?Co=Y (accessed March 24, 2020).
29. NHS Scotland. eHealth. HEPMA. Available from: https://www.ehealth.scot/case-studies/hepma/ (accessed March 24, 2020).
30. Scottish Intensive Care Society Audit Group. Available from: https://www.sicsag.scot.nhs.uk/about/main.html (accessed March 30, 2020).
31. Health Protection Scotland. Data and surveillance. Available from: https://www.hps.scot.nhs.uk/data/ (accessed March 25, 2020).
32. NHS Greater Glasgow and Clyde. West of Scotland Specialist Virology Centre. Available from: https://www.nhsggc.org.uk/about-us/professional-support-sites/laboratory-medicine/laboratory-disciplines/microbiology-and-virology/west-of-scotland-specialist-virology-centre/# (accessed March 25, 2020).
33. Victora CG, Huttly SR, Fuchs SC, Olinto MT. The role of conceptual frameworks in epidemiological analysis: a hierarchical approach. *Int J Epidemiol* 1997; 26(1): 224-7.

Figure 1. Flow diagram for EAVE II project.

For peer review only - http://bmjopen.bmj.com/site/about/guidelines.xhtml

ECOSS: Electronic Communication of Surveillance in Scotland; NHS: National Health Service; HEPMA: Hospital Electronic Prescribing and Medicines Administration; RT-PCR: reverse transcriptase polymerase chain reaction; PHS: Public Health Scotland; eDRIS: The electronic Data Research and Innovation Service; ISD: Information Services Scotland.

For peer review only

For peer review only - http://bmjopen.bmj.com/site/about/guidelines.xhtml

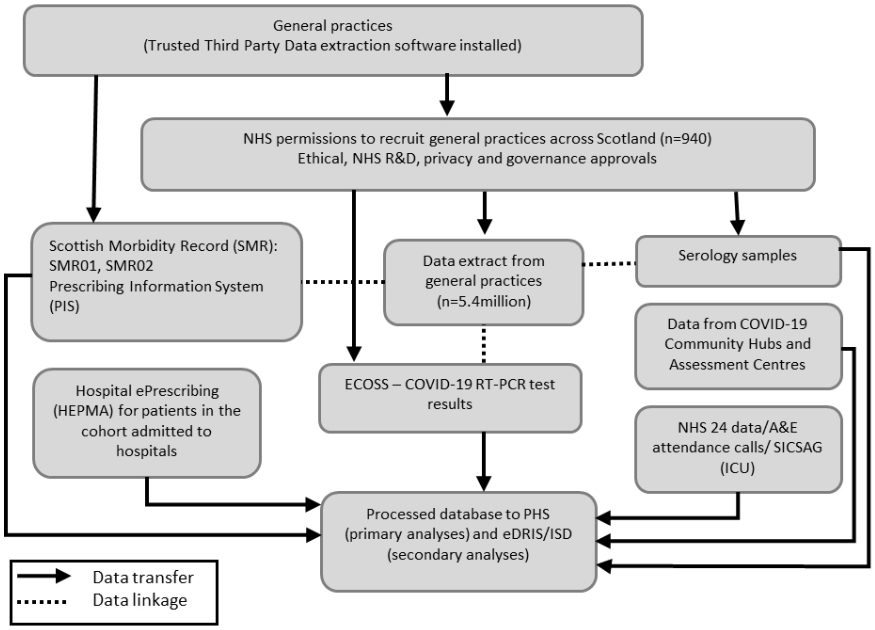

Figure 1. Flow diagram for EAVE II project.
ECOSS: Electronic Communication of Surveillance in Scotland; NHS: National Health Service; HEPMA: Hospital Electronic Prescribing and Medicines Administration; RT-PCR: reverse transcriptase polymerase chain reaction; PHS: Public Health Scotland; eDRIS: The electronic Data Research and Innovation Service; ISD: Information Services Scotland.

90x90mm (300 x 300 DPI)
